# The Trp64Arg polymorphism in β3 adrenergic receptor (*ADRB3*) gene is associated with adipokines and plasma lipids: a systematic review, meta-analysis, and meta-regression

**DOI:** 10.1186/s12944-020-01290-y

**Published:** 2020-05-19

**Authors:** Zhi Luo, Ting Zhang, Shengping Wang, Yuxian He, Qiutang Ye, Wenzhai Cao

**Affiliations:** 1 Department of Cardiology, The First People’s Hospital of Zigong, Zigong, 643000 People’s Republic of China; 2 Department of Nursing, Sichuan Vocational College of Health and Rehabilitation, Zigong, 643000 People’s Republic of China; 3 North Sichuan Medical College, Nanchong, 637000 People’s Republic of China; 4 Office of Research Affairs, The First People’s Hospital of Zigong, Zigong, 643000 People’s Republic of China

**Keywords:** *ADRB3*, Trp64Arg, Polymorphism, Adipokines, Plasma lipids, CAD, Obesity

## Abstract

**Background:**

Recently, some studies claim that adipokines may modulate plasma lipids. More interestingly, the *ADRB3* Trp64Arg polymorphism may regulate adipokines and play an essential role in lipids metabolism. This study aims to clarify the associations of *ADRB3* Trp64Arg polymorphism with plasma adipokines and lipid levels.

**Methods:**

Twenty-two studies (5527 subjects) and 121 studies (54,059 subjects) were respectively identified for the association analyses of adipokines and lipids. Standardized mean difference (SMD) and 95% confidence interval (CI) were used to estimate the strength of the Trp64Arg variant in adipokines and plasma lipids. All results were recalculated after eliminating the studies with heterogeneity.

**Results:**

The carriers of the C allele (Arg at 64th position was encoded by the C allele) had higher levels of leptin and lower levels of adiponectin than the non-carriers. The carriers of the C allele had higher levels of triglycerides (TG), total cholesterol (TC), and lower levels of high-density lipoprotein cholesterol (HDL-C) than the non-carriers. Subgroup analysis certified an ethnicity (Asians), disease status (obesity), and gender (females) specific association. Sensitivity analysis indicated that the analysis results were robust and stable. Meta-regression indicated that obesity was related to adiponectin.

**Conclusions:**

The C allele carriers of Trp64Arg polymorphism had a slight but significant influence on lipid levels, and the remarkable effects specific existed in obese Asian women. The associations of Trp64Arg polymorphism with dyslipidemia may partly be mediated by the effect of this polymorphism on adipokines. The association of Trp64Arg polymorphism with obesity may partly be mediated by the effect of this polymorphism on adipokines. The C allele carriers had abnormal levels of adipokines and lipids, and it indicated that the Trp64Arg polymorphism might represent a genetic risk factor for coronary artery disease (CAD).

## Introduction

CAD obtains the highest mortality and disability rate in most of the developed and developing countries. CAD is triggered by multiple genetic and environmental risk factors. Among these risk factors, the abnormal levels of adipokines [1, 2] and lipids [3] are widely reported about their essential roles in the occurrence and progress of CAD. Both leptin and adiponectin are called adipokines, produced by white adipose tissue, one of the most potent lipid regulator [4–6], and plays a vital role in lipid metabolism [7–9]. Some new shreds of evidence have shown that leptin is positively related to dyslipidemia [10–12] in contrast to adiponectin, which is negatively related to dyslipidemia [13–15]. Considering the leptin is considerably increased, and the adiponectin is substantially decreased in obese mice [16]. Leptin promotes, and adiponectin prevents atherosclerosis [17] by aiming directly on blood vessel endothelial cells. The adipokines are becoming an indispensable bridge between obesity, dyslipidemia, and atherosclerosis [18–20]. As the leptin/adiponectin ratio serves as a new atherosclerotic indicator [21], it is a sensitive index in estimating obesity and dyslipidemia.

The beta-adrenergic receptors (ADRBs) are composed of the β1 adrenergic receptor (ADRB1), β2 adrenergic receptor (ADRB2), and ADRB3. The ADRB1 and ADRB2 located in myocytes and play an essential role in its function regulation [22, 23]. By contrast, the ADRB3 subtype located in white adipose tissue and plays a critical role in regulating lipolysis in white adipose tissue [24, 25]. The *ADRB3* gene is located on the long arm of human chromosome 8 (8p11.1–12), and it contains two exons and one intron. The Trp64Arg polymorphism (also known as T190C, W64R, and rs4994) is the only function mutation of the *ADRB3* gene, located in exon one and caused by substitution from thymine (T) to cytosine (C). Previous studies have reported that the *ADRB3* gene might regulate the expression levels of adipokines [26, 27] and plasma lipids [28, 29]. Also, the Trp64Arg polymorphism was proved to be related to obesity [30–32] and relevant characteristics [33, 34] in Pima Indians and western obese patients. Whereas the *ADRB3* gene might influence the plasma levels of adipokines and lipids and the Trp64Arg polymorphism might be associated with obesity-related traits, it is not difficult to speculate that the Trp64Arg variant might influence the plasma levels of adipokines and lipids. Recently, Chen et al. [35] have reported that the Trp64Arg variant is closely related to CAD. However, the specific mechanisms are not precise, so there is an urgent need to clarify the associations of Trp64Arg polymorphism with plasma adipokines and lipid levels, to provide some clues or references for the clarification of possible mechanisms of Trp64Arg polymorphism with CAD.

It is worth noting that this is the first systematic review, meta-analysis, and meta-regression to assess the associations of the Trp64Arg polymorphism with adipokines and plasma lipids in a large sample size (59,586 subjects of 122 studies). Besides, the present systematic review may generate some new information. Some studies have reported that the Trp64Arg variant was positively associated with adipokines and plasma lipids. On the contrary, other studies have indicated that the Trp64Arg variant was negatively associated with adipokines and plasma lipids. However, the results from a few studies did not support these associations. Since the present study results are controversial and inconclusive, a systematic review, meta-analysis, and meta-regression are required to unveil whether this polymorphism affects adipokines and plasma lipids or not. Moreover, if it does, so whether it is positive or negative in the light of evidence-based medicine.

## Methods

### Literature search

A comprehensive literature review was performed before Jan 2020 by using nine databases including PubMed, Cochrane Library, Medline, Embase, Web of Science, Google Scholar, Wanfang, China National Knowledge Infrastructure (CNKI), and China Biology Medicine (CBM) database by using Keywords: (“ADRB3” or “beta3-adrenergic receptor” or “β3-adrenergic receptor” or “β3AR”), (“Trp64Arg” or “T190C” or “rs4994”), (“polymorphism” or “mutation” or “variant”), (“adipokines”), (“adiponectin”), (“leptin”) and (“circulating lipid” or “blood lipid” or “plasma lipid” or “serum lipid”).

### Inclusion criteria

The inclusion criteria for this systematic review are as below: (1) The studies have examined the associations of Trp64Arg polymorphism with adipokines and plasma lipids. (2) The studies have at least offered one of the variables in adipokines profile (leptin and adiponectin) or lipids profile [TG, TC, low-density lipoprotein cholesterol (LDL-C), and HDL-C]. (3) The studies have offered the mean adipokines or lipids with standard deviation (SD) by genotypes. (4) The studies have provided the allele or genotype frequencies of the Trp64Arg polymorphism. (5) The language of included studies is restricted to English and Chinese. The exclusion criteria for this study are as below: (1) Studies not related to Trp64Arg polymorphism; (2) studies do not offer genotype or allele frequencies; (3) studies present invalid data; (4) pedigree studies; (5) overlapping studies; (6) abstract, review, systematic review, meta-analysis, and animal studies.

### Data extraction

According to a pre-piloted data extraction form and the pre-specified selection criteria, two researchers independently extracted the data from each eligible study. Results were compared, and divergences about data extraction were settled by consensus. If crucial data were absent from included studies, e-mail, or telephone would be used to acquire these data. From each eligible study, the following data were extracted: the first author’s name, the publication year, language, country, ethnicity, disease status, gender, genotype counts, total sample size, and mean plasma levels of adiponectin, leptin, and lipids with SD by genotypes.

### Data analysis

All the tests were performed by STATA software (version 15.0, College Station, TX). *P*-values less than 0.05 were considered to be statistically significant. The Chi-square test estimated the Hardy–Weinberg equilibrium (HWE) among control subjects. Publication bias was tested by Begg’s funnel plot and Egger’s test [36]. SMD with 95% CIs were used to assess the effects of the Trp64Arg variant on plasma adipokines and lipid levels. Sensitivity analysis was used to check the robustness and stableness of all results. The random-effect model was performed to analyze the results if heterogeneity among the involved studies was remarkable. Otherwise, the Fixed-effect model would be used [37]. Galbraith plots and meta-regression analysis estimated the sources of heterogeneity among studies. All SMD with 95% CIs were recalculated after excluding the study with heterogeneities. The confounding factors included publication year (before 2000 and after 2000)), language (English and Chinese), ethnicity (Caucasian, Asian, Chilean, Brazilian, Indonesian and Other ethnic), gender (Males or Females), disease status [Obesity, CAD, type 2 diabetes mellitus (T2DM), and Hypertension] and total sample size (≥500 subjects and < 500 subjects). Subgroup analyses were conducted by ethnicity, disease status, gender, healthy subjects, and children subjects. The ethnic subgroup was divided into Caucasian, Asian, Chilean, Brazilian, Indonesian, and other ethnicities. Disease status was divided into Obesity, CAD, T2DM, and Hypertension.

## Result

### Characteristics of the included studies

**Fig. 1:**
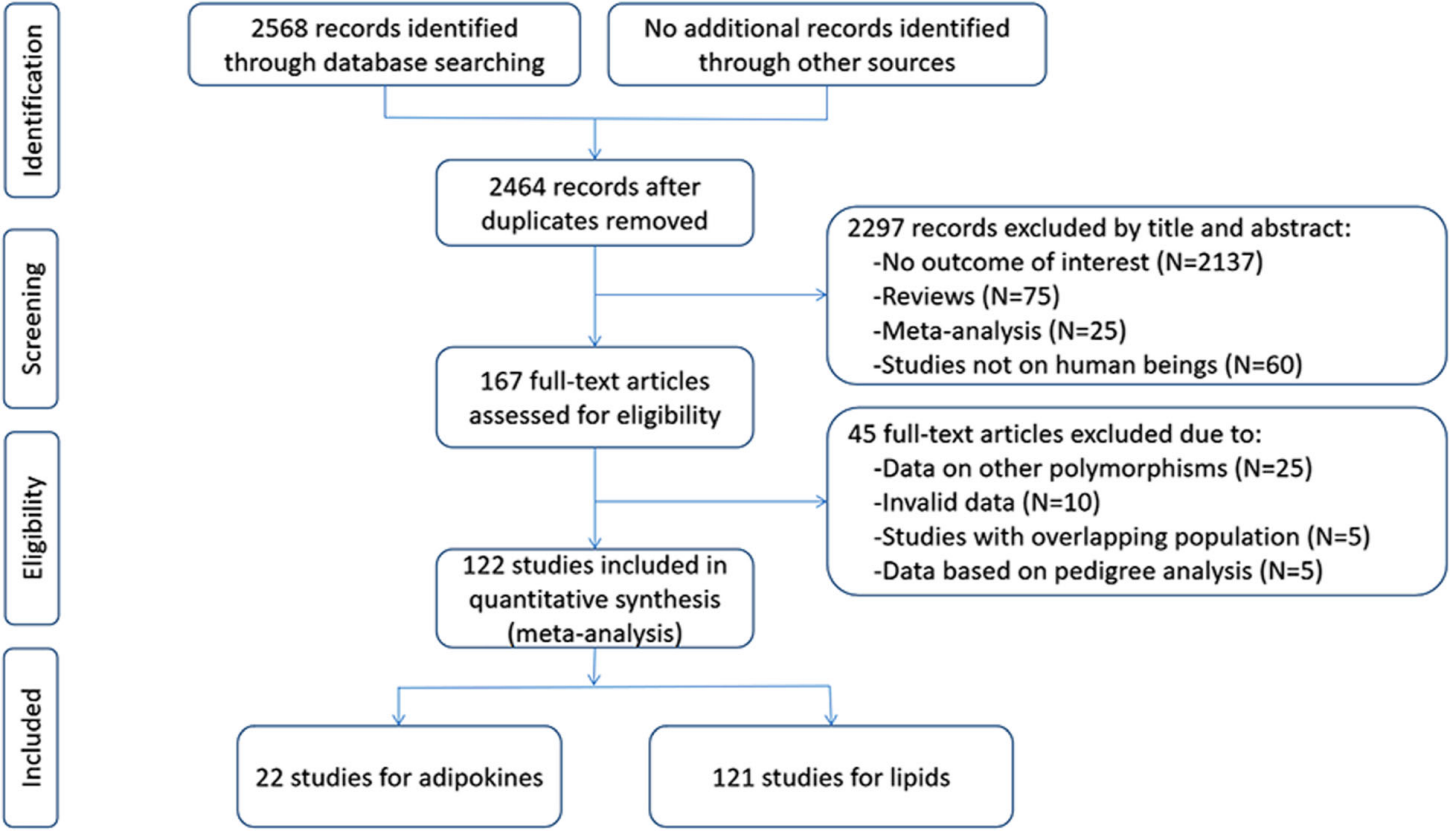
Flow diagram of the study selection process

The initial search of the databases yielded 2568 studies. Two thousand two hundred and ninety-seven studies were excluded by its contents. Then 167 studies were reevaluated by the inclusion criteria. Forty-five studies were further excluded due to the following reasons: twenty-five studies offered data of other polymorphisms, ten studies offered invalid data, five studies had subjects overlapping with other publications, and five studies were based on pedigree analysis. Finally, 122 studies were included for this systematic review (Fig. 1). Of them, 22 studies (5527 subjects) and 121 studies (54,059 subjects) were identified for the association analyses of adipokines and lipids, respectively.

The references of the included studies were listed in Additional file 1. The characteristics of the included studies were presented in Additional file 1: Table S1. The plasma adipokines and lipid levels by the genotypes of the *ADRB3* Trp64Arg polymorphism were presented in Additional file 1: Table S2 and Additional file 1: Table S3, respectively. The sources of heterogeneity of TG, HDL-C, adiponectin, leptin, TC and LDL-C were respectively presented in Additional file 1: Table S4, Additional file 1: Table S5, Additional file 1: Table S6, Additional file 1: Table S7, Additional file 1: Table S8 and Additional file 1: Table S9.

### Association of the *ADRB3* Trp64Arg polymorphism with plasma adipokines levels

**Fig. 2:**
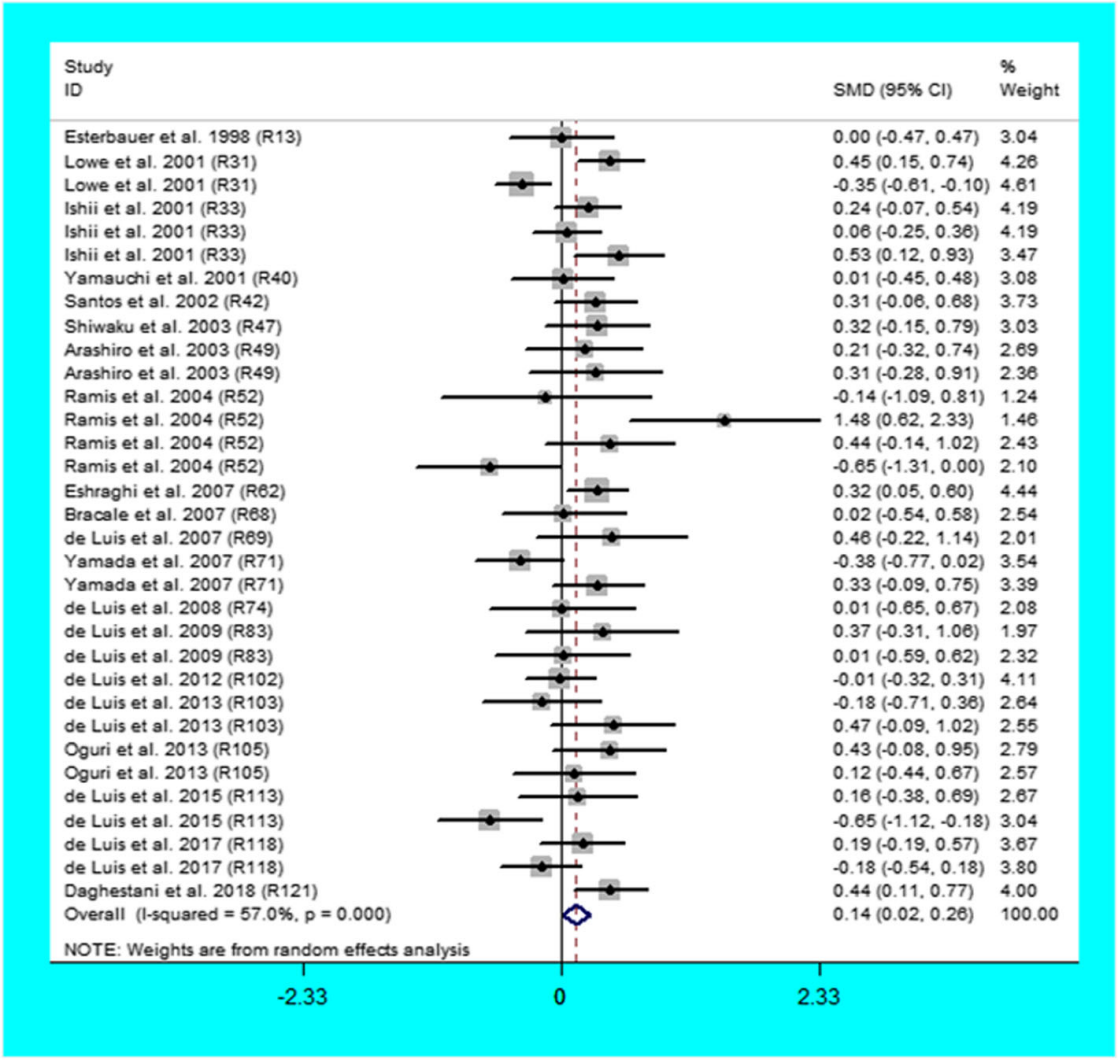
Forest plot of the meta-analysis between the *ADRB3* Trp64Arg polymorphism and leptin

**Fig. 3:**
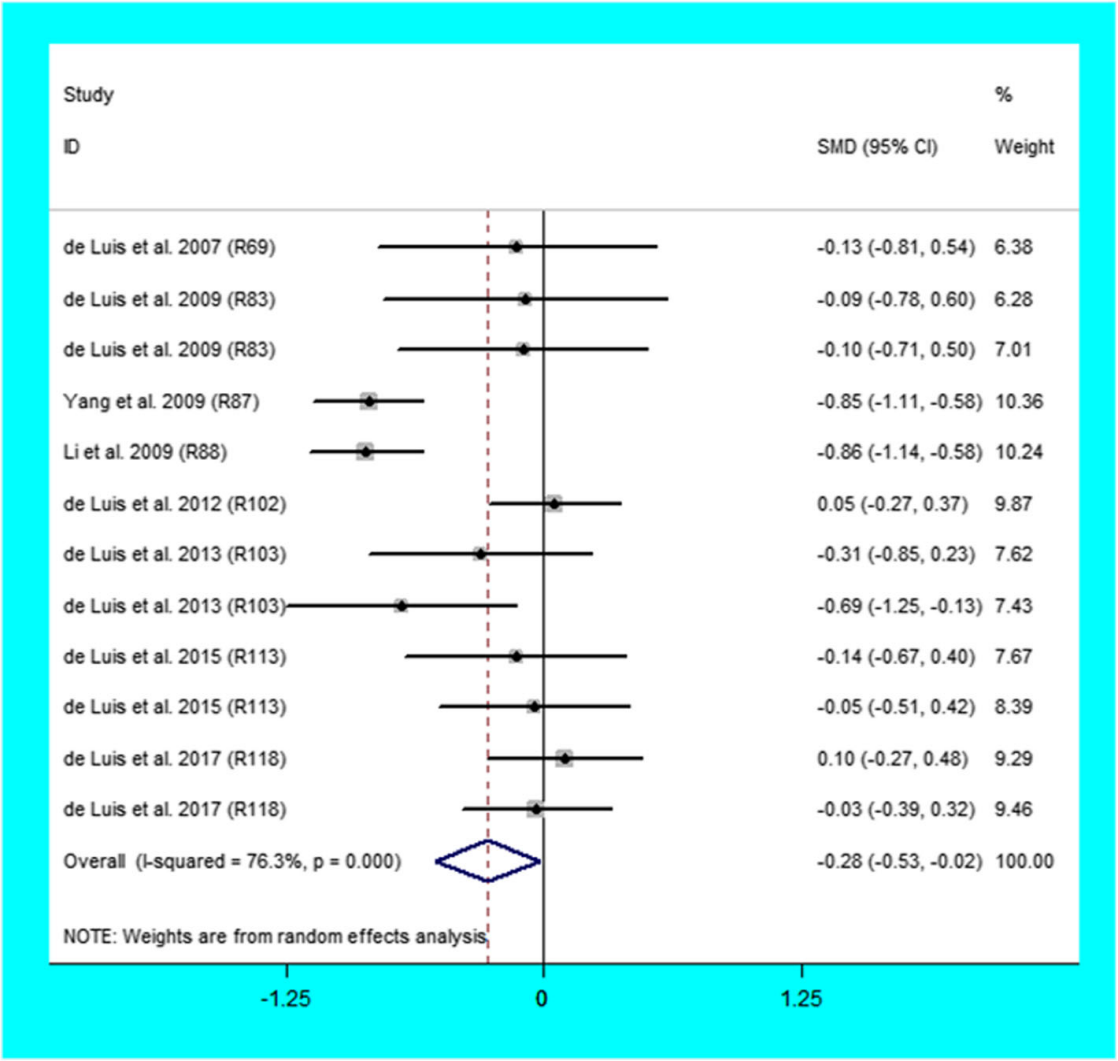
Forest plot of the meta-analysis between the *ADRB3* Trp64Arg polymorphism and adiponectin

The C allele carriers had higher levels of leptin (Fig. 2) and lower levels of adiponectin (Fig. 3) than the non-carriers. When the analysis was confined to the studies in HWE (Table 1), the significant association of the Trp64Arg polymorphism with higher levels of leptin and lower levels of adiponectin were also detected.

**Table 1:** Meta-analysis of the *ADRB3* Trp64Arg polymorphism with adipokines levels

Groups or subgroups	Comparisons (Subjects)	SMD (95% CI)	*P*_Heterogeneity_	*P*_SMD_
Leptin				
All	33 (4999)	0.14 (0.02–0.26)	< 0.001	0.02
Studies in HWE	31 (4594)	0.12 (0.00–0.24)	< 0.001	0.05
Caucasian	19 (3036)	0.11 (− 0.06–0.28)	< 0.01	0.21
Asian	11 (1124)	0.18 (0.03–0.33)	0.18	0.02
Female	9 (1416)	0.11 (−0.18–0.41)	< 0.001	0.46
Male	9 (1161)	0.22 (0.02–0.42)	0.04	0.03
Obesity	19 (2418)	0.08 (−0.09–0.24)	0.01	0.36
Healthy subjects	12 (2384)	0.19 (0.00–0.38)	< 0.001	0.05
Adiponectin				
All	12 (2073)	−0.28 (−0.53--0.02)	< 0.001	0.03
Studies in HWE	11 (1800)	− 0.26 (− 0.39--0.13)	< 0.001	< 0.001
Caucasian	10 (1545)	− 0.08 (− 0.23–0.07)	0.64	0.28
Asian	2 (528)	−0.86 (−1.05--0.66)	0.95	< 0.001
Obesity	10 (1545)	− 0.08 (− 0.23–0.07)	0.64	0.28

*ADRB3*: beta3-adrenergic receptor gene, SMD: standardized mean difference, 95% CI: 95% confidence interval, *HWE* Hardy-Weinberg equilibrium

Then the subgroup analyses were conducted (Table 1), and the analyses revealed that the Trp64Arg variant was related to higher levels of leptin in Asians, Males, and healthy subjects and lower levels of adiponectin in Asians.

The analyses eliminating the studies with heterogeneity were also conducted (Table 2), and the analyses showed that the Trp64Arg variant was related to higher levels of leptin in Caucasians, Asians, Males, and healthy subjects and lower levels of adiponectin in Caucasians and Asians.

**Table 2:** Meta-analysis of the *ADRB3* Trp64Arg polymorphism with adipokines levels after excluding the study with heterogeneity

Groups or subgroups	Comparisons (Subjects)	SMD (95% CI)	*P*_Heterogeneity_	*P*_SMD_
Leptin				
All	29 (4581)	0.17 (0.09–0.24)	0.08	< 0.001
Studies in HWE	27 (4176)	0.14 (0.06–0.23)	0.07	< 0.001
Caucasian	16 (2733)	0.16 (0.05–0.27)	0.46	0.01
Asian	10 (1009)	0.24 (0.11–0.37)	0.80	< 0.001
Female	7 (1253)	−0.02 (− 0.17–0.12)	0.05	0.76
Male	8 (1046)	0.30 (0.16–0.44)	0.52	< 0.001
Obesity	16 (2113)	0.10 (−0.02–0.22)	0.83	0.11
Healthy subjects	11 (2271)	0.19 (0.09–0.30)	0.06	< 0.001
Adiponectin				
All	8 (1190)	− 0.63 (− 0.78--0.48)	0.07	< 0.001
Studies in HWE	7 (917)	−0.52 (− 0.70--0.34)	0.10	< 0.001
Caucasian	6 (662)	−0.26 (− 0.50--0.02)	0.68	0.03
Asian	2 (528)	−0.86 (−1.05--0.66)	0.95	< 0.001
Obesity	6 (662)	−0.08 (− 0.23–0.07)	0.64	0.28

*ADRB3* beta3-adrenergic receptor gene, *SMD* standardized mean difference, *95% CI* 95% confidence interval, *HWE* Hardy-Weinberg equilibrium

### Association of the *ADRB3* Trp64Arg polymorphism with plasma lipid levels

The C allele carriers had higher levels of TG and TC and lower levels of HDL-C than the non-carriers (Table 3). When the analysis was confined to the studies in HWE (Table 3), the significant association of the Trp64Arg polymorphism with higher levels of TG, TC, and lower levels of HDL-C were also detected.

**Table 3:** Meta-analysis of the *ADRB3* Trp64Arg polymorphism with plasma lipid levels

Groups or subgroups	Comparisons (Subjects)	SMD (95% CI)	*P*_Heterogeneity_	*P*_SMD_
TG				
All	162 (43,778)	0.07 (0.03–0.11)	< 0.001	< 0.001
Studies in HWE	142 (38,507)	0.05 (0.02–0.09)	< 0.001	0.01
Caucasian	47 (17,065)	0.09 (0.02–0.16)	< 0.01	< 0.01
Asian	101 (24,424)	0.06 (0.02–0.11)	< 0.01	< 0.01
Chilean	3 (340)	0.04 (−0.41–0.50)	0.02	0.85
Brazilian	2 (223)	0.22 (−0.13–0.57)	0.37	0.22
Indonesian	4 (531)	−0.18 (− 0.45–0.09)	0.84	0.20
Other ethnic	5 (1195)	0.14 (−0.16–0.45)	< 0.01	0.35
Female	40 (10,329)	0.06 (0.01–0.11)	0.01	0.01
Male	30 (9499)	0.03 (−0.02–0.08)	< 0.01	0.30
Obesity	38 (5772)	0.12 (0.05–0.18)	0.32	< 0.01
T2DM	20 (4335)	0.03 (− 0.01–0.07)	< 0.001	0.07
Hypertension	4 (781)	−0.05 (− 0.22–0.13)	0.28	0.61
CAD	7 (1225)	−0.12 (− 0.25–0.01)	0.46	0.07
Healthy subjects	69 (25,873)	0.05 (0.00–0.10)	< 0.001	0.03
Children	14 (2337)	0.12 (−0.05–0.29)	< 0.001	0.16
TC				
All	162 (49,738)	0.04 (0.01–0.07)	< 0.001	0.01
Studies in HWE	140 (43,716)	0.03 (0.01–0.05)	< 0.001	0.01
Caucasian	46 (19,238)	0.07 (0.00–0.13)	< 0.01	0.04
Asian	104 (27,572)	0.02 (0.00–0.05)	< 0.001	0.05
Chilean	3 (340)	0.04 (−0.19–0.27)	0.83	0.73
Brazilian	4 (1310)	0.09 (−0.06–0.24)	0.52	0.23
Other ethnic	5 (1278)	0.00 (−0.27–0.27)	< 0.01	0.98
Female	38 (11,568)	0.06 (0.02–0.11)	0.23	0.01
Male	29 (9543)	−0.00 (− 0.08–0.08)	< 0.01	0.92
Obesity	36 (5400)	0.07 (0.00–0.13)	0.32	0.04
T2DM	20 (5257)	0.04 (−0.02–0.11)	0.75	0.22
Hypertension	6 (1182)	0.04 (− 0.10–0.18)	0.57	0.54
CAD	7 (1225)	−0.11 (− 0.27–0.06)	0.28	0.20
Healthy subjects	68 (27,441)	0.05 (0.01–0.10)	< 0.001	0.02
Children	14 (2279)	0.03 (−0.13–0.20)	< 0.01	0.67
LDL-C				
All	103 (25,965)	0.03 (−0.02–0.07)	< 0.001	0.24
Studies in HWE	86 (21,459)	−0.01 (− 0.05–0.04)	0.01	0.82
Caucasian	29 (7687)	0.01 (−0.06–0.07)	0.07	0.88
Asian	64 (15,264)	0.04 (−0.02–0.09)	< 0.001	0.22
Brazilian	4 (1310)	0.02 (− 0.35–0.38)	0.02	0.94
Other ethnic	5 (1338)	0.07 (−0.17–0.30)	0.01	0.59
Female	24 (5800)	0.05 (− 0.02–0.11)	0.30	0.17
Male	19 (3140)	−0.04 (− 0.17–0.08)	0.01	0.51
Obesity	30 (4930)	0.01 (−0.06–0.08)	0.36	0.81
T2DM	10 (2345)	0.17 (− 0.06–0.40)	< 0.001	0.14
CAD	6 (1040)	−0.05 (− 0.18–0.09)	0.87	0.51
Healthy subjects	39 (12,149)	0.05 (−0.02–0.12)	< 0.001	0.20
Children	11 (2080)	0.10 (−0.09–0.30)	< 0.001	0.30
HDL-C				
All	164 (49,069)	−0.05 (− 0.08--0.02)	< 0.001	< 0.01
Studies in HWE	144 (43,919)	−0.04 (− 0.07--0.01)	< 0.001	0.03
Caucasian	45 (17,720)	−0.06 (− 0.08--0.04)	0.60	0.05
Asian	102 (27,648)	−0.05 (− 0.09--0.01)	< 0.001	0.02
Chilean	3 (340)	−0.10 (− 0.33–0.13)	0.37	0.38
Brazilian	4 (1310)	− 0.14 (− 0.33–0.04)	0.62	0.22
Indonesian	4 (531)	−0.07 (− 0.34–0.20)	0.52	0.59
Other ethnic	4 (613)	−0.32 (− 0.58--0.07)	0.13	0.01
Female	40 (12,250)	− 0.04 (− 0.08--0.00)	0.22	0.05
Male	31 (9407)	−0.01 (− 0.08–0.07)	0.01	0.91
Obesity	36 (5310)	−0.06 (− 0.10--0.02)	< 0.01	0.04
T2DM	19 (3575)	− 0.17 (− 0.30--0.04)	< 0.01	0.01
Hypertension	4 (957)	0.01 (− 0.15–0.16)	0.10	0.91
CAD	8 (1532)	−0.14 (− 0.37–0.09)	0.01	0.23
Healthy subjects	72 (27,600)	−0.00 (− 0.03–0.03)	0.16	0.93
Children	15 (2409)	−0.09 (− 0.18–0.00)	0.06	0.06

*ADRB3* beta3-adrenergic receptor gene, *SMD* standardized mean difference, *95% CI* 95% confidence interval, *HWE* Hardy-Weinberg equilibrium, *TG* triglycerides, *TC* total cholesterol, *LDL-C* low-density lipoprotein cholesterol, *HDL-C* high-density lipoprotein cholesterol, *CAD* coronary artery disease, *T2DM* type 2 diabetes mellitus

Then the subgroup analysis by the characteristics of the subjects was performed (Table 3). Subgroup analysis by ethnicity showed that the significant associations of the Trp64Arg polymorphism with higher levels of TG, TC, and lower levels of HDL-C were detected in Caucasians and Asians, but not in the other ethnicities. Subgroup analysis by gender showed that the significant associations of the Trp64Arg polymorphism with higher levels of TG, TC, and lower levels of HDL-C were only detected in the females, but not in the males. Subgroup analysis by disease status showed that the significant associations of the Trp64Arg polymorphism with higher levels of TG and TC were only detected in obese patients, but not in CAD patients, T2DM patients, and hypertension patients. Besides, the significant association of the Trp64Arg polymorphism with lower levels of HDL-C was detected in obese patients and T2DM patients, but not in CAD patients and hypertension patients. When the analysis was limited to healthy subjects, the significant association of the Trp64Arg polymorphism with higher levels of TG and TC were also detected.

The analyses eliminating the studies with heterogeneity were also conducted (Table 4), and the analyses revealed that the Trp64Arg variant was related to higher levels of TG, TC, and lower levels of HDL-C in Asians, females and obesity patients after excluding the outlier studies. However, the association analysis of the Trp64Arg variant with lipid levels did not show statistically significant in Caucasians and healthy subjects. Besides, the significant association of the Trp64Arg variant with higher levels of LDL-C was detected in Asians. This indicated that the significant associations of the Trp64Arg variant with plasma lipid levels in obese Asian women were stable and robust.

**Table 4:** Meta-analysis of the *ADRB3* Trp64Arg polymorphism with plasma lipid levels after excluding the study with heterogeneity

Groups or subgroups	Comparisons (Subjects)	SMD (95% CI)	*P*_Heterogeneity_	*P*_SMD_
TG				
All	146 (38,535)	0.04 (0.01–0.06)	0.07	< 0.01
Studies in HWE	130 (34,679)	0.02 (0.00–0.05)	0.24	0.05
Caucasian	42 (14,786)	0.04 (−0.01–0.09)	0.37	0.08
Asian	92 (21,766)	0.04 (0.01–0.07)	0.05	0.01
Chilean	2 (234)	−0.18 (− 0.47–0.10)	0.79	0.21
Brazilian	2 (223)	0.22 (−0.13–0.57)	0.37	0.22
Indonesian	4 (531)	−0.18 (− 0.45–0.09)	0.84	0.20
Other ethnic	4 (995)	−0.05 (− 0.19–0.09)	0.28	0.51
Female	38 (10,163)	0.05 (0.00–0.10)	0.05	0.05
Male	26 (7602)	0.01 (−0.05–0.06)	0.30	0.83
Obesity	37 (5290)	0.09 (0.02–0.16)	0.49	0.01
T2DM	17 (3592)	0.13 (0.05–0.21)	0.42	< 0.01
Hypertension	4 (781)	−0.05 (− 0.22–0.13)	0.28	0.61
CAD	7 (1225)	− 0.12 (− 0.25–0.01)	0.46	0.07
Healthy	62 (22,922)	0.02 (−0.02–0.05)	0.08	0.35
Children	11 (2025)	0.08 (−0.01–0.18)	0.68	0.08
TC				
All	153 (46,775)	003 (0.01–0.05)	0.11	< 0.01
Studies in HWE	133 (41,641)	0.03 (0.01–0.06)	0.17	0.01
Caucasian	42 (17,507)	0.04 (−0.01–0.08)	0.43	0.11
Asian	99 (26,340)	0.03 (0.01–0.06)	0.20	0.01
Chilean	3 (340)	0.04 (−0.19–0.27)	0.83	0.73
Brazilian	4 (1310)	0.09 (−0.06–0.24)	0.52	0.23
Other ethnic	5 (1278)	0.00 (−0.27–0.27)	< 0.01	0.98
Female	36 (11,377)	0.05 (0.00–0.10)	0.76	0.04
Male	25 (7681)	−0.01 (− 0.06–0.05)	0.34	0.81
Obesity	35 (5269)	0.08 (0.00–0.15)	0.70	0.04
T2DM	20 (5257)	0.04 (−0.02–0.11)	0.75	0.22
Hypertension	6 (1182)	0.04 (−0.10–0.18)	0.57	0.54
CAD	7 (1225)	−0.13 (− 0.26–0.00)	0.28	0.06
Healthy	62 (25,355)	0.03 (−0.00–0.06)	0.13	0.07
Children	12 (2039)	0.04 (−0.05–0.14)	0.39	0.36
LDL-C				
All	93 (22,397)	0.02 (−0.01–0.05)	0.54	0.16
Studies in HWE	81 (19,151)	0.02 (−0.02–0.05)	0.49	0.38
Caucasian	27 (7116)	−0.01 (− 0.08–0.06)	0.49	0.84
Asian	58 (12,539)	0.04 (0.00–0.08)	0.52	0.04
Brazilian	3 (1238)	−0.03 (− 0.19–0.12)	0.20	0.67
Other ethnic	4 (1138)	−0.07 (− 0.20–0.06)	0.85	0.31
Female	23 (5740)	0.04 (−0.03–0.10)	0.66	0.29
Male	17 (2512)	0.01 (−0.08–0.10)	0.12	0.84
Obesity	29 (4688)	0.02 (−0.05–0.09)	0.57	0.55
T2DM	8 (1890)	0.03 (−0.08–0.14)	0.44	0.62
CAD	6 (1040)	−0.05 (− 0.18–0.09)	0.87	0.51
Healthy	34 (9862)	0.05 (−0.00–0.09)	0.22	0.07
Children	8 (1768)	0.10 (−0.00–0.20)	0.42	0.06
HDL-C				
All	150 (43,951)	−0.04 (− 0.06--0.02)	0.55	0.01
Studies in HWE	134 (39,781)	−0.03 (− 0.06--0.01)	0.22	0.01
Caucasian	45 (17,720)	−0.04 (− 0.09–0.00)	0.60	0.06
Asian	90 (23,675)	−0.03 (− 0.06--0.01)	0.05	0.02
Chilean	3 (340)	−0.10 (− 0.33–0.13)	0.37	0.38
Brazilian	3 (295)	0.21 (−0.09–0.50)	0.42	0.17
Indonesian	4 (531)	−0.07 (− 0.34–0.20)	0.52	0.59
Other ethnic	3 (483)	−0.23 (− 042--0.04)	0.55	0.02
Female	38 (11,946)	−0.05 (− 0.10--0.00)	0.73	0.03
Male	27 (8774)	−0.02 (− 0.07–0.03)	0.13	0.50
Obesity	33 (5017)	−0.05 (− 0.10--0.00)	0.17	0.05
T2DM	15 (1923)	−0.15 (− 0.25--0.05)	0.68	< 0.01
Hypertension	4 (957)	0.01 (−0.15–0.16)	0.10	0.91
CAD	7 (1403)	−0.07 (− 0.19–0.06)	0.09	0.31
Healthy	67 (24,960)	−0.02 (− 0.05–0.01)	0.49	0.21
Children	14 (2279)	−0.07 (− 0.15–0.02)	0.33	0.15

*ADRB3* beta3-adrenergic receptor gene, *SMD* standardized mean difference, *95% CI* 95% confidence interval, *HWE* Hardy-Weinberg equilibrium, *TG* triglycerides, *TC* total cholesterol, *LDL-C* low-density lipoprotein cholesterol, *HDL-C* high-density lipoprotein cholesterol, *CAD* coronary artery disease, *T2DM* type 2 diabetes mellitus

### Evaluation of heterogeneity

In the association analysis of adipokines, significant heterogeneity was identified in the total comparisons of leptin and adiponectin (Table 1). Four and four comparisons were recognized as the main contributors to the heterogeneity for leptin and adiponectin, respectively, by performing Galbraith plots. The analysis results of leptin and adiponectin did not change substantially after excluding the outlier comparisons (Table 2).

In the association analysis of lipids, significant heterogeneity was identified in the total comparisons for TG, TC, LDL-C, and HDL-C (Table 3). Sixteen, nine, ten, and eleven comparisons were respectively recognized as the main contributors to the heterogeneity for TG, TC, LDL-C, HDL-C, by performing Galbraith plots. The analysis results of lipids did not change substantially after excluding the outlier comparisons (Table 4).

Univariate and multivariate meta-regression analysis was also conducted to explore sources of heterogeneity among the included studies. Furthermore, the analyses revealed that the total sample size (Table S4, Table S5) was a significant source of heterogeneity of TG (*P*_univariate_ = 0.05) and HDL-C (*P*_univariate_ < 0.01, *P*_multivariate_ = 0.01). Also, the disease status (Table S6) was a significant source of heterogeneity of adiponectin (*P*_univariate_ < 0.001, *P*_multivariate_ < 0.001). However, no confounding factors (Table S7-S9) explain the heterogeneity of leptin (*P*-values 0.51 to 0.98), TC (*P*-values 0.37 to 0.85), and LDL-C (*P*-values 0.10 to 0.82).

### Sensitivity analyses

In association analysis of the Trp64Arg polymorphism with plasma adipokines and lipids levels, a sensitivity analysis was conducted by calculating all results again after omitting every single study. Interestingly, the analysis results of adipokines and plasma lipids did not change substantially after omitting these studies, which indicated that the analysis results were robust and stable.

### Publication bias test

Begg’s test and Egger’s test were used to evaluating the publication bias of the included studies, and no publication bias was detected (*P* = 0.90 for leptin, 0.68 for adiponectin, 0.89 for TG, 0.28 for TC, 0.39 for LDL-C, 0.37 for HDL-C, respectively).

## Discussion

The present study showed that the *ADRB3* Trp64Arg polymorphism is robustly associated with abnormal levels of adipokines and lipids. This indicates that the *ADRB3* Trp64Arg polymorphism may represent a genetic risk factor for CAD.

The specific mechanisms in which the *ADRB3* Trp64Arg polymorphism with abnormal adipokines have not been clarified yet. One possible mechanism can be proposed to explain the relationship between the Trp64Arg variant and abnormal adipokines levels: The mutation C allele of *ADRB3* results in low mRNA expression levels [38] and low protein activity [39] of hormone-sensitive lipase (HSL), the reduce lipase concentration and protein activity will no doubt initiate or accelerate obesity [30–32]. Interestingly, this hypothesis is verified in this meta-analysis, since the data shows that the mutation C allele of *ADRB3* is robustly associated with obesity (Table 3, Table 4), as described above, the obesity is related to abnormal levels of adipokines [16]. This may explain the present findings.

Several possible mechanisms can be proposed to explain the relationship between the *ADRB3* Trp64Arg variant and dyslipidemia. At first, HSL can promote the free fatty acid and glycerol release from white adipose tissue [40, 41] into the plasma, and the changed lipase concentration and protein activity caused by C allele [38, 39] will no doubt influence the plasma concentration of free fatty acid and glycerol, thereby induced dyslipidemia. Secondly, Li et al. [42] have conducted an animal study in 40 apolipoprotein E (*APOE*) gene knock-out mice. Their data suggest that the *ADRB3* gene may trigger dyslipidemia by regulating the expression of proprotein convertase subtilisin/kexin type 9 (PCSK9) gene and LDL receptor (LDLR) gene. It is widely known that both PCSK9 and LDLR are lipid metabolism-regulated genes, thereby the Trp64Arg polymorphism may indirectly affect plasma lipids levels by regulating lipid metabolism-regulated genes expression. Thirdly, whereas both leptin [10–12] and adiponectin [13–15] are related to dyslipidemia, the Trp64Arg variant may indirectly affect lipids levels through the mediation of adipokines. Interestingly, this hypothesis may also verify in this meta-analysis. The data in the present study shows that the mutation C allele of Trp64Arg polymorphism only has a slight effect on plasma levels of lipids (Table 3, Table 4), but a strong effect on plasma levels of adipokines (Table 1, Table 2). Whose SMD values are much larger than those calculated in plasma lipids. As mentioned above, adipokines as a lipid regulator [4–6] play a critical role in lipid metabolism [7–9]. This may also explain the present findings.

In the present meta-analysis, the significant effects of the Trp64Arg variant on lipid levels were only in Asians (Table 4), it indicates that there is an interaction between the Trp64Arg variant and ethnicity in modulating the plasma lipids. Gender might modulate the association of the Trp64Arg variant with plasma lipids since the significant effects of the Trp64Arg variant on lipid levels were only in Females (Table 4). Disease status might also modulate the association of the Trp64Arg variant with plasma lipids since the significant effects of the Trp64Arg variant on lipid levels were mainly from patients with obesity (Table 3, Table 4). Besides, whose SMD values were much smaller than those calculated in adipokines (Table 1, Table 2). When combined with the previous findings [43], it indicated that the association of the Trp64Arg variant with obesity might partly be mediated by the effect of this variant on adipokines.

### Strengths and limitations

Several strengths of the present meta-analysis should be put forward. Firstly, the present meta-analysis had the sufficiently high statistical power to examine the associations of the Trp64Arg polymorphism with adipokines and plasma lipids in a large sample size, which would increase the reliability of the calculated results. Secondly, the calculated results were obtained after excluding the studies with heterogeneity, which would undoubtedly contribute to drawing some scientific and precise conclusions in the present meta-analysis. Thirdly, the multi-level analyses were performed by ethnicity, disease status, gender, healthy subjects, and children subjects, which would undoubtedly be beneficial to generate some comprehensive and diversified results in this present study. The main limitation of the present meta-analysis was that multiple genetic and environmental factors triggered dyslipidemia. However, the interactions between Trp64Arg polymorphism and other genetic or environmental factors on plasma lipids have not been investigated in this work due to the lack of original data.

## Conclusions

The C allele carriers of Trp64Arg polymorphism had a slight but significant influence on lipid levels, and the remarkable effects specific existed in obese Asian women. The associations of Trp64Arg polymorphism with dyslipidemia may partly be mediated by the effect of this polymorphism on adipokines. The association of Trp64Arg polymorphism with obesity may partly be mediated by the effect of this polymorphism on adipokines. The C allele carriers had abnormal levels of adipokines and lipids, and it indicated that the Trp64Arg polymorphism might represent a genetic risk factor for CAD.

## Supplementary information

**Additional file 1 Table S1.** Characteristics of the included studies in this systematic review of plasma adipokines and lipids levels for the *ADRB3* Trp64Arg polymorphism. **Table S2.** Plasma adipokines levels by the genotypes of the *ADRB3* Trp64Arg polymorphism. **Table S3.** Plasma lipids levels by the genotypes of the *ADRB3* Trp64Arg polymorphism. **Table S4.** Meta-regression analysis explores the sources of heterogeneity of plasma triglycerides (TG) levels. **Table S5.** Meta-regression analysis explores the sources of heterogeneity of plasma high-density lipoprotein cholesterol (HDL-C) levels. **Table S6.** Meta-regression analysis explores the sources of heterogeneity of plasma adiponectin levels. **Table S7.** Meta-regression analysis explores the sources of heterogeneity of plasma leptin levels. **Table S8.** Meta-regression analysis explores the sources of heterogeneity of plasma total cholesterol (TC) levels. **Table S9.** Meta-regression analysis explores the sources of heterogeneity for circulating low-density lipoprotein cholesterol (LDL-C) levels. **Supplementary References:** The reference list of the included studies in this systematic review.

## Abbreviations

ADRB3: β3 adrenergic receptor
CAD: Coronary artery disease
T2DM: Type 2 diabetes mellitus
TG: Triglycerides
TC: Total cholesterol
LDL-C: Low-density lipoprotein cholesterol
HDL-C: High-density lipoprotein cholesterol
HSL: Hormone-sensitive lipase
SMD: Standardized mean difference
95% CI: 95% confidence interval
SD: Standard deviation
APOE: Apolipoprotein E
PCSK9: Proprotein convertase subtilisin/kexin type 9
LDLR: LDL receptor
CNKI: China National Knowledge Infrastructure
CBM: China Biology Medicine
HWE: Hardy-Weinberg equilibrium
