# Supplementary material for: The Trp64Arg polymorphism in β3 adrenergic receptor (ADRB3) gene is associated with adipokines and plasma lipids: a systematic review, meta-analysis, and meta-regression
Source: Lipids Health Dis. 2020 May 19;19:99. doi: 10.1186/s12944-020-01290-y (PMC7236936; doi:10.1186/s12944-020-01290-y)
Supplement: Supplementary file 1 — Additional file 1 Table S1. Characteristics of the included studies in this systematic review of plasma adipokines and lipids levels for the ADRB3 Trp64Arg polymorphism. Table S2. Plasma adipokines levels by the genotypes of the ADRB3 Trp64Arg polymorphism. Table S3. Plasma lipids levels by the genotypes of the ADRB3 Trp64Arg polymorphism. Table S4. Meta-regression analysis explores the sources of heterogeneity of plasma triglycerides (TG) levels. Table S5. Meta-regression analysis explores the sources of heterogeneity of plasma high-density lipoprotein cholesterol (HDL-C) levels. Table S6. Meta-regression analysis explores the sources of heterogeneity of plasma adiponectin levels. Table S7. Meta-regression analysis explores the sources of heterogeneity of plasma leptin levels. Table S8. Meta-regression analysis explores the sources of heterogeneity of plasma total cholesterol (TC) levels. Table S9. Meta-regression analysis explores the sources of heterogeneity for circulating low-density lipoprotein cholesterol (LDL-C) levels. Supplementary References: The reference list of the included studies in this systematic review. [file 12944_2020_1290_MOESM1_ESM.doc]

**Supplemental Tables:**

**Table S1.** Characteristics of the included studies in this systematic review of plasma adipokines and lipids levels for the *ADRB3* Trp64Arg polymorphism.

**Table S2.** Plasma adipokines levels by the genotypes of the *ADRB3* Trp64Arg polymorphism.

**Table S3.** Plasma lipids levels by the genotypes of the *ADRB3* Trp64Arg polymorphism.

**Table S4.** Meta-regression analysis explore the sources of heterogeneity of plasma triglycerides (TG) levels.

**Table S5.** Meta-regression analysis explore the sources of heterogeneity of plasma high-density lipoprotein cholesterol (HDL-C) levels.

**Table S6.** Meta-regression analysis explore the sources of heterogeneity of plasma adiponectin levels.

**Table S7.** Meta-regression analysis explore the sources of heterogeneity of plasma leptin levels.

**Table S8.** Meta-regression analysis explore the sources of heterogeneity of plasma total cholesterol (TC) levels.

**Table S9.** Meta-regression analysis explore the sources of heterogeneity for circulating low-density lipoprotein cholesterol (LDL-C) levels.

**Table S1.** Characteristics of the included studies in this systematic review of plasma adipokines and lipids levels for the *ADRB3* Trp64Arg polymorphism.

| **First author, reference** | **year** | **Ethnicity** | **Gender** | **Study population** | **Outcomes** |
| --- | --- | --- | --- | --- | --- |
| Elbein SC [R1] | 1996 | Caucasian | M/F | T2DM patients | TG |
| Urhammer SA [R2] | 1996 | Caucasian | M/F | Healthy subjects | TG, TC, LDL-C, HDL-C |
| Higashi K [R3] | 1997 | Asian | M/F | CAD patients | TG, TC, LDL-C, HDL-C |
| Fujisawa T [R4] | 1997 | Asian | M/F | Hypertension patients | TC |
| Sakane N1 [R5] | 1997 | Asian | F | Obesity patients | TG, TC, HDL-C |
| Sakane N2 [R5] | 1997 | Asian | F | Healthy subjects | TG, TC, HDL-C |
| Yuan XH1 [R6] | 1997 | Asian | M | Healthy subjects | TG, TC, HDL-C |
| Yuan XH2 [R6] | 1997 | Asian | F | Healthy subjects | TG, TC, HDL-C |
| Sakane N3 [R7] | 1997 | Asian | M/F | T2DM patients | TG, TC, HDL-C |
| Rissanen J1 [R8] | 1997 | Caucasian | M/F | T2DM patients | TG, TC, HDL-C |
| Rissanen J2 [R8] | 1997 | Caucasian | M/F | Insulin resistance patients | TG, TC, HDL-C |
| Rissanen J3 [R8] | 1997 | Caucasian | M/F | Healthy subjects | TG, TC, HDL-C |
| ARII K1 [R9] | 1997 | Asian | M | T2DM patients | TG, TC, HDL-C |
| ARII K2 [R9] | 1997 | Asian | F | T2DM patients | TG, TC, HDL-C |
| ARII K3 [R9] | 1997 | Asian | M | Healthy subjects | TG, TC, HDL-C |
| ARII K4 [R9] | 1997 | Asian | F | Healthy subjects | TG, TC, HDL-C |
| Sakane N4 [R10] | 1998 | Asian | M/F | T2DM patients | TG, TC, HDL-C |
| Buettner R1 [R11] | 1998 | Caucasian | M | Healthy subjects | TC, HDL-C |
| Buettner R2 [R11] | 1998 | Caucasian | F | Healthy subjects | TC, HDL-C |
| Sun L1 [R12] | 1998 | Asian | M | Healthy subjects | TG, TC, LDL-C, HDL-C |
| Sun L2 [R12] | 1998 | Asian | F | Healthy subjects | TG, TC, LDL-C, HDL-C |
| Sun L3 [R12] | 1998 | Asian | M | T2DM/Hypertension patients | TG, TC, LDL-C, HDL-C |
| Sun L4 [R12] | 1998 | Asian | F | T2DM/Hypertension patients | TG, TC, LDL-C, HDL-C |
| Esterbauer H [R13] | 1998 | Caucasian | M/F | Obesity patients | TG, TC, HDL-C, Leptin |
| Shima Y [R14] | 1998 | Asian | M/F | Healthy subjects | TG, TC, HDL-C |
| Pulkkinen A1 [R15] | 1999 | Caucasian | M/F | CAD patients | TG, TC, HDL-C |
| Pulkkinen A2 [R15] | 1999 | Caucasian | M/F | CAD/T2DM patients | TG, TC, HDL-C |
| Ghosh S [R16] | 1999 | Caucasian | M/F | T2DM/Obesity patients | TG, TC, HDL-C |
| Kawamura T [R17] | 1999 | Asian | M | Healthy subjects | TG, TC, HDL-C |
| Tonolo G1 [R18] | 1999 | Caucasian | M/F | Hypertension patients | TG, TC, LDL-C, HDL-C |
| Tonolo G2 [R18] | 1999 | Caucasian | M/F | Healthy subjects | TG, TC, LDL-C, HDL-C |
| Hayakawa T [R19] | 1999 | Asian | M | Healthy subjects | TG, TC, HDL-C |
| Sheu WH1 [R20] | 1999 | Asian | M/F | CAD patients | TG, TC, LDL-C, HDL-C |
| Sheu WH2 [R20] | 1999 | Asian | M/F | Healthy subjects | TG, TC, LDL-C, HDL-C |
| Festa A [R21] | 1999 | Caucasian | F | Gestational diabetes mellitus patients and control subjects | TG, TC |
| Kikuchi T1 [R22] | 2000 | Asian | M/F | Obesity patients (Children) | TG, TC, HDL-C |
| Kikuchi T2 [R22] | 2000 | Asian | M/F | Obesity patients (Children) | TG, TC, HDL-C |
| Kikuchi T3 [R22] | 2000 | Asian | M/F | Obesity patients (Children) | TG, TC, HDL-C |
| Bao AM1 [R23] | 2000 | Asian | M/F | T2DM patients | TG, TC, HDL-C |
| Bao AM2 [R23] | 2000 | Asian | M/F | Healthy subjects | TG, TC, HDL-C |
| Sun LM1 [R24] | 2000 | Asian | M | T2DM patients and control subjects | TG, TC, LDL-C, HDL-C |
| Sun LM2 [R24] | 2000 | Asian | F | T2DM patients and control subjects | TG, TC, LDL-C, HDL-C |
| Sun LM3 [R24] | 2000 | Asian | M | T2DM patients and control subjects | TG, TC, LDL-C, HDL-C |
| Sun LM4 [R24] | 2000 | Asian | F | T2DM patients and control subjects | TG, TC, LDL-C, HDL-C |
| Thomas GN [R25] | 2000 | Asian | M/F | Metabolic syndrome patients/Healthy subjects | TC, LDL-C |
| Strazzullo P [R26] | 2000 | Caucasian | M | Healthy subjects | TG, TC |
| Urhammer SA [R27] | 2000 | Caucasian | M/F | Healthy subjects | TG, TC, LDL-C, HDL-C |
| Benecke H [R28] | 2000 | Caucasian | F | Obesity patients | TG, TC |
| Pamies-Andreu E [R29] | 2000 | Caucasian | M/F | Hypertension patients | TG, TC, LDL-C, HDL-C |
| Endo K [R30] | 2000 | Asian | M/F | Healthy subjects (Children) | TG, TC, LDL-C, HDL-C |
| Lowe WL Jr1 [R31] | 2001 | Other ethnic | M | Healthy subjects | Leptin |
| Lowe WL Jr2 [R31] | 2001 | Other ethnic | F | Healthy subjects | Leptin |
| Corella D1 [R32] | 2001 | Caucasian | M | Healthy subjects | TG, TC, LDL-C, HDL-C |
| Corella D2 [R32] | 2001 | Caucasian | F | Healthy subjects | TG, TC, LDL-C, HDL-C |
| Ishii T1 [R33] | 2001 | Asian | M | Healthy subjects | TG, TC, LDL-C, HDL-C, Leptin |
| Ishii T2 [R33] | 2001 | Asian | M | Healthy subjects | TG, TC, LDL-C, HDL-C, Leptin |
| Ishii T3 [R33] | 2001 | Asian | M | Hyperlipidemia patients | TG, TC, LDL-C, HDL-C, Leptin |
| Chen Y1 [R34] | 2001 | Asian | M/F | T2DM patients | TG, TC, HDL-C |
| Chen Y2 [R34] | 2001 | Asian | M/F | Healthy subjects | TG, TC, HDL-C |
| Wang XL [R35] | 2001 | Asian | M/F | Healthy subjects (Children) | TG, TC, LDL-C, HDL-C |
| Chen Y3[R36] | 2001 | Asian | M/F | Obesity patients | TG, TC, HDL-C |
| Chen Y4 [R36] | 2001 | Asian | M/F | Healthy subjects | TG, TC, HDL-C |
| Carlsson M [R37] | 2001 | Caucasian | M/F | T2DM patients and control subjects | TG, TC, HDL-C |
| Oizumi T [R38] | 2001 | Asian | M/F | Healthy subjects | TG, TC, HDL-C |
| Manraj M [R39] | 2001 | Other ethnic | M/F | CAD patients | HDL-C |
| Yamauchi T [R40] | 2001 | Asian | M/F | Haemodialysis patients | TG, HDL-C, Leptin |
| Wang GY [R41] | 2002 | Asian | M/F | T2DM patients | TG, TC, HDL-C |
| Santos JL [R42] | 2002 | Other ethnic | M/F | Healthy subjects | TG, TC, HDL-C, Leptin |
| Matsushita H1 [R43] | 2003 | Asian | F | Healthy subjects | TG, TC, LDL-C, HDL-C |
| Matsushita H2 [R43] | 2003 | Asian | F | Healthy subjects | TG, TC, LDL-C, HDL-C |
| Matsushita Y1 [R44] | 2003 | Asian | M | Healthy subjects | TC, HDL-C |
| Matsushita Y2 [R44] | 2003 | Asian | F | Healthy subjects | TC, HDL-C |
| Zhu LY1 [R45] | 2003 | Asian | M/F | Healthy subjects | TG, TC, LDL-C, HDL-C |
| Zhu LY2 [R45] | 2003 | Asian | M/F | Overweight patients | TG, TC, LDL-C, HDL-C |
| Zhu LY3 [R45] | 2003 | Asian | M/F | Obesity patients | TG, TC, LDL-C, HDL-C |
| Tan N1 [R46] | 2003 | Asian | M/F | CAD patients | TG, TC, LDL-C, HDL-C |
| Tan N2 [R46] | 2003 | Asian | M/F | Healthy subjects | TG, TC, LDL-C, HDL-C |
| Shiwaku K [R47] | 2003 | Asian | F | Healthy subjects | TG, TC, LDL-C, HDL-C, Leptin |
| Okumura K [R48] | 2003 | Asian | M/F | Healthy subjects | TG, TC, LDL-C, HDL-C |
| Arashiro R1 [R49] | 2003 | Asian | M | Obesity patients | TG, TC, LDL-C, HDL-C, Leptin |
| Arashiro R2 [R49] | 2003 | Asian | F | Obesity patients | TG, TC, LDL-C, HDL-C, Leptin |
| Kim OY [R50] | 2004 | Asian | M/F | Obesity patients | TG, TC, LDL-C, HDL-C |
| Gao CR1 [R51] | 2004 | Asian | M | T2DM patients | TG, TC, LDL-C, HDL-C |
| Gao CR2 [R51] | 2004 | Asian | F | T2DM patients | TG, TC, LDL-C, HDL-C |
| Ramis JM1 [R52] | 2004 | Caucasian | M | Obesity patients | TG, TC, LDL-C, HDL-C, Leptin |
| Ramis JM2 [R52] | 2004 | Caucasian | F | Obesity patients | TG, TC, LDL-C, HDL-C, Leptin |
| Ramis JM3 [R52] | 2004 | Caucasian | M | Healthy subjects | TG, TC, LDL-C, HDL-C, Leptin |
| Ramis JM4 [R52] | 2004 | Caucasian | F | Healthy subjects | TG, TC, LDL-C, HDL-C, Leptin |
| Erhardt E [R53] | 2005 | Other ethnic | M/F | Obesity patients (Children) | TG, TC, HDL-C |
| Zuo H [R54] | 2005 | Asian | M/F | T2DM patients | TG, TC, HDL-C |
| Tan H1 [R55] | 2005 | Asian | M/F | Hypertension patients | TG, TC |
| Tan H2 [R55] | 2005 | Asian | M/F | Healthy subjects | TG, TC |
| Miyaki K [R56] | 2005 | Asian | M | Healthy subjects | TG, TC, HDL-C |
| Perez-Bravo F1 [R57] | 2005 | Other ethnic | F | Polycystic ovary syndrome patients | TG, TC, HDL-C |
| Perez-Bravo F2 [R57] | 2005 | Other ethnic | F | Healthy subjects | TG, TC, HDL-C |
| Lee JS [R58] | 2006 | Asian | F | Overweight patients | TG, TC, LDL-C, HDL-C |
| Kim K1 [R59] | 2006 | Asian | F | Obesity patients | TG, TC, LDL-C, HDL-C |
| Kim K2 [R59] | 2006 | Asian | F | Healthy subjects | TG, TC, LDL-C, HDL-C |
| Yuan M [R60] | 2006 | Asian | M/F | Hypertension patients | TC, HDL-C |
| Tamaki S1 [R61] | 2006 | Asian | M | Healthy subjects | TG, HDL-C |
| Tamaki S2 [R61] | 2006 | Asian | F | Healthy subjects | TC, HDL-C |
| Eshraghi P [R62] | 2007 | Caucasian | M/F | Healthy subjects | TG, TC, HDL-C, Leptin |
| Klass DM [R63] | 2007 | Caucasian | M/F | Gallstone patients | TG, TC, HDL-C |
| Li Q [R64] | 2007 | Asian | M/F | Obesity patients (Children) | TG, TC, LDL-C, HDL-C |
| Jiang XY1 [R65] | 2007 | Asian | M | T2DM patients | TG, TC, LDL-C, HDL-C |
| Jiang XY2 [R65] | 2007 | Asian | F | T2DM patients | TG, TC, LDL-C, HDL-C |
| Chen W1 [R66] | 2007 | Caucasian | M/F | Healthy subjects | LDL-C |
| Chen W2 [R66] | 2007 | Other ethnic | M/F | Healthy subjects | LDL-C |
| Pierola J [R67] | 2007 | Caucasian | M/F | Obstructive sleep apnoea syndrome patients | TG, TC, HDL-C |
| Bracale R [R68] | 2007 | Caucasian | M/F | Obesity patients | TG, LDL-C, HDL-C, Leptin |
| de Luis DA1 [R69] | 2007 | Caucasian | M/F | Obesity patients | TG, TC, LDL-C, HDL-C, Leptin, Adiponectin |
| Ueno T [R70] | 2007 | Asian | M/F | Healthy subjects | TG, TC, HDL-C |
| Dunajska K [R71] | 2007 | Caucasian | F | Healthy subjects | TG, TC, LDL-C, HDL-C |
| Yamada K1 [R72] | 2007 | Asian | M | Obesity patients | TG, TC, LDL-C, HDL-C, Leptin |
| Yamada K2 [R72] | 2007 | Asian | F | Obesity patients | TG, TC, LDL-C, HDL-C, Leptin |
| Tsuzakia K [R73] | 2007 | Asian | M/F | Healthy subjects | TG, TC, HDL-C |
| de Luis DA2 [R74] | 2008 | Caucasian | M/F | Obesity patients | TG, TC, LDL-C, HDL-C, Leptin |
| Kotani K [R75] | 2008 | Asian | M/F | Healthy subjects | TG, TC, LDL-C, HDL-C |
| Chen H [R76] | 2008 | Asian | M/F | Hypertension patients and control subjects | TG, TC, LDL-C, HDL-C |
| Zhu LY4 [R77] | 2008 | Asian | M/F | Obesity patients | TG, TC, LDL-C, HDL-C |
| Gjesing AP1 [R78] | 2008 | Caucasian | M | Healthy subjects | TG, TC, HDL-C |
| Gjesing AP2 [R78] | 2008 | Caucasian | F | Healthy subjects | TG, TC, HDL-C |
| Nonen S [R79] | 2008 | Asian | M/F | T2DM patients | TG, TC, LDL-C, HDL-C |
| Hamada T [R80] | 2008 | Asian | F | Healthy subjects | TG, TC, LDL-C, HDL-C |
| Dunajska K [R81] | 2008 | Caucasian | F | Healthy subjects | TG, TC, LDL-C, HDL-C |
| Zafarmand MH [R82] | 2008 | Caucasian | F | Healthy subjects | TC, LDL-C, HDL-C |
| de Luis DA3 [R83] | 2009 | Caucasian | M/F | Obesity patients | TG, TC, LDL-C, HDL-C, Leptin, Adiponectin |
| de Luis DA4 [R83] | 2009 | Caucasian | M/F | Obesity patients | TG, TC, LDL-C, HDL-C, Leptin, Adiponectin |
| Zhu LY5 [R84] | 2009 | Asian | M/F | Obesity patients | TG, TC, LDL-C, HDL-C |
| Yu B [R85] | 2009 | Asian | M/F | Hypertension patients and control subjects | TC, HDL-C |
| Zhang JH [R86] | 2009 | Asian | M/F | Obesity patients and control subjects (Children) | TG, TC, LDL-C, HDL-C |
| Yang M [R87] | 2009 | Asian | M/F | T2DM patients | TG, TC, LDL-C, HDL-C, Adiponectin |
| Li HH [R88] | 2009 | Asian | M/F | T2DM patients | TG, TC, LDL-C, HDL-C, Adiponectin |
| Chen YZ1 [R89] | 2010 | Asian | M/F | CAD patients | TG, TC, LDL-C, HDL-C |
| Chen YZ2 [R89] | 2010 | Asian | M/F | CAD patients | TG, TC, LDL-C, HDL-C |
| Zhu LY6 [R90] | 2010 | Asian | M/F | Obesity patients | TG, TC, LDL-C, HDL-C |
| Zhu LY7 [R90] | 2010 | Asian | M/F | Healthy subjects | TG, TC, LDL-C, HDL-C |
| Kim SM1 [R91] | 2010 | Asian | M | Healthy subjects | TG, TC, LDL-C, HDL-C |
| Kim SM2 [R91] | 2010 | Asian | M | Healthy subjects | TG, TC, LDL-C, HDL-C |
| de Luis DA5 [R92] | 2010 | Caucasian | M/F | Obesity patients | TG, TC, LDL-C, HDL-C |
| Morcillo S [R93] | 2010 | Caucasian | M/F | Healthy subjects | TG, TC |
| Peng AN [R94] | 2010 | Asian | M/F | Obesity patients (Children) | TG, TC, LDL-C, HDL-C |
| Genelhu VA [R95] | 2010 | Other ethnic | M/F | Obesity patients | TG, TC, LDL-C, HDL-C |
| Tsunekawa K [R96] | 2011 | Asian | M/F | Healthy subjects | TG, TC, HDL-C |
| Mirrakhimov AE [R97] | 2011 | Other ethnic | M/F | Healthy subjects | TC, LDL-C, HDL-C |
| Malik SG1 [R98] | 2011 | Other ethnic | M | Healthy subjects | TG, HDL-C |
| Malik SG2 [R98] | 2011 | Other ethnic | F | Healthy subjects | TG, HDL-C |
| Malik SG3 [R98] | 2011 | Other ethnic | M | Healthy subjects | TG, HDL-C |
| Malik SG4 [R98] | 2011 | Other ethnic | F | Healthy subjects | TG, HDL-C |
| Iwamoto Y [R99] | 2011 | Asian | M/F | Hypertension patients | TG, TC, HDL-C |
| Kwon DH [R100] | 2012 | Asian | F | Obesity patients | TG, TC, HDL-C |
| Zhang XY [R101] | 2012 | Other ethnic | M/F | Hypertension patients and control subjects | TG, TC, LDL-C |
| de Luis DA6 [R102] | 2012 | Caucasian | M/F | Obesity patients | TG, TC, LDL-C, HDL-C, Leptin, Adiponectin |
| de Luis DA7 [R103] | 2013 | Caucasian | M/F | Obesity patients | TG, TC, LDL-C, HDL-C, Leptin, Adiponectin |
| de Luis DA8 [R103] | 2013 | Caucasian | M/F | Obesity patients | TG, TC, LDL-C, HDL-C, Leptin, Adiponectin |
| Hameed I [R104] | 2013 | Other ethnic | M/F | T2DM patients | TG, TC, LDL-C, HDL-C |
| Oguri K1 [R105] | 2013 | Asian | M | Healthy subjects (Children) | TG, TC, LDL-C, HDL-C, Leptin |
| Oguri K2 [R105] | 2013 | Asian | F | Healthy subjects (Children) | TG, TC, LDL-C, HDL-C, Leptin |
| Zhang XL [R106] | 2014 | Asian | M | Metabolic syndrome patients (Children) | TG, TC, LDL-C, HDL-C |
| Kumar S [R107] | 2014 | Other ethnic | M/F | CAD patients | TG, TC, LDL-C, HDL-C |
| Brondani LA [R108] | 2014 | Other ethnic | M/F | T2DM patients | TC, LDL-C, HDL-C |
| Iemitsu M [R109] | 2014 | Asian | M/F | Healthy subjects | TG, TC, HDL-C |
| Wang T [R110] | 2014 | Asian | M/F | Healthy subjects | TG, TC, LDL-C, HDL-C |
| Yoshihara A [R111] | 2014 | Asian | F | Healthy subjects | TG, TC |
| Hui ZG [R112] | 2014 | Asian | M/F | T2DM patients | TG, TC, LDL-C, HDL-C |
| de Luis DA9 [R113] | 2015 | Caucasian | M/F | Obesity patients | TG, TC, LDL-C, HDL-C, Leptin, Adiponectin |
| de Luis DA10 [R113] | 2015 | Caucasian | M/F | Obesity patients | TG, TC, LDL-C, HDL-C, Leptin, Adiponectin |
| Chen YH1 [R114] | 2015 | Asian | M/F | Obesity patients | TG, TC, LDL-C, HDL-C |
| Chen YH2 [R114] | 2015 | Asian | M/F | Healthy subjects | TG, TC, LDL-C, HDL-C |
| Verdi H [R115] | 2015 | Other ethnic | M/F | Obesity patients (Children) | TG, LDL-C, HDL-C |
| Liu JL [R116] | 2015 | Asian | M/F | Healthy subjects | TG, TC, LDL-C, HDL-C |
| Dong CM [R117] | 2016 | Asian | M | Metabolic syndrome patients | TG, TC, LDL-C, HDL-C |
| de Luis DA11 [R118] | 2017 | Caucasian | F | Obesity patients | TG, TC, LDL-C, HDL-C, Leptin, Adiponectin |
| de Luis DA12 [R118] | 2017 | Caucasian | F | Obesity patients | TG, TC, LDL-C, HDL-C, Leptin, Adiponectin |
| Jesus IC [R119] | 2018 | Other ethnic | M/F | Healthy subjects (Children) | TC, LDL-C, HDL-C |
| Milano-Gai GE [R120] | 2018 | Other ethnic | M/F | Obesity patients (Children) | TG, TC, LDL-C, HDL-C |
| Daghestani M [R121] | 2018 | Caucasian | M/F | Healthy subjects | TG, TC, LDL-C, HDL-C, Leptin |
| Grygiel-Gorniak B1 [R122] | 2018 | Caucasian | F | Healthy subjects | TG, HDL-C |
| Grygiel-Gorniak B2 [R122] | 2018 | Caucasian | F | Healthy subjects | TG, HDL-C |

*ADRB3*: beta3-adrenergic receptor gene; M: male; F: female; CAD: coronary artery disease; T2DM: type 2 diabetes mellitus; TG: triglycerides; TC: total cholesterol; LDL-C: low-density lipoprotein cholesterol; HDL-C: high-density lipoprotein cholesterol

**Table S2.** Plasma adipokines levels by the genotypes of the *ADRB3* Trp64Arg polymorphism. Data are expressed as M±SD.

| **First author, reference** | **Genotype** | |  | **Leptin, ng/ml** | |  | **Adiponectin, ng/ml** | |
| --- | --- | --- | --- | --- | --- | --- | --- | --- |
|  | **TT** | **TC+CC** |  | **TT** | **TC+CC** |  | **TT** | **TC+CC** |
| Esterbauer H [R13] | 133 | 20 |  | 36.0±17.7 | 36.0±17.3 |  | - | - |
| Lowe WL Jr1 [R31] | 210 | 56 |  | 6.7±6.6 | 11.2±18.0 |  | - | - |
| Lowe WL Jr2 [R31] | 347 | 74 |  | 31.1±24.5 | 22.8±18.0 |  | - | - |
| Ishii T1 [R33] | 137 | 59 |  | 2.5±2.0 | 3.05±2.92 |  | - | - |
| Ishii T2 [R33] | 125 | 61 |  | 3.7±1.7 | 3.8±2 |  | - | - |
| Ishii T3 [R33] | 89 | 33 |  | 3.7±1.4 | 4.5±1.8 |  | - | - |
| Yamauchi T [R40] | 46 | 29 |  | 14.4±18.99 | 14.58±12.75 |  | - | - |
| Santos JL [R42] | 114 | 38 |  | 9.8±10.2 | 14.6±25.6 |  | - | - |
| Shiwaku K [R47] | 49 | 27 |  | 7.2±4.1 | 10.3±15.3 |  | - | - |
| Arashiro R1 [R49] | 34 | 23 |  | 10.8±4.08 | 12.0±7.67 |  | - | - |
| Arashiro R2 [R49] | 31 | 17 |  | 13.9±6.12 | 16.0±7.83 |  | - | - |
| Ramis JM1 [R52] | 30 | 5 |  | 8.31±5.11 | 7.62±3.66 |  | - | - |
| Ramis JM2 [R52] | 43 | 7 |  | 26.5±11.4 | 43.7±13.3 |  | - | - |
| Ramis JM3 [R52] | 99 | 13 |  | 3.99±2.6 | 5.19±3.47 |  | - | - |
| Ramis JM4 [R52] | 103 | 10 |  | 12.9±7.6 | 8.03±5.72 |  | - | - |
| Eshraghi P [R62] | 340 | 61 |  | 23±21 | 30±25 |  | - | - |
| Bracale R [R68] | 229 | 13 |  | 110.9±63.0 | 112.1±54.8 |  |  |  |
| de Luis DA1 [R69] | 55 | 10 |  | 59.8±28 | 73±33 |  | 23.6±17 | 21.3±19 |
| Yamada K1 [R71] | 78 | 37 |  | 8.88±6.28 | 6.79±3.59 |  | - | - |
| Yamada K2 [R72] | 87 | 30 |  | 20.4±9.98 | 24.07±13.87 |  | - | - |
| de Luis DA2 [R74] | 45 | 11 |  | 167.6±66 | 168±57 |  | - | - |
| de Luis DA3 [R83] | 87 | 9 |  | 109.1±65 | 133.4±64 |  | 25.6±26.9 | 23.1±36 |
| de Luis DA4 [R83] | 85 | 12 |  | 122.1±64 | 123±66 |  | 27.2±21.2 | 25.0±25.0 |
| Yang M [R87] | 187 | 86 |  | - | - |  | 3.15±2.12 | 1.57±1.10 |
| Li HH [R88] | 178 | 77 |  | - | - |  | 3.20±2.10 | 1.60±1.10 |
| de Luis DA6 [R102] | 162 | 50 |  | 103.2±90.1 | 102.7±40 |  | 40.7±17.0 | 41.5±11.2 |
| de Luis DA7 [R103] | 112 | 15 |  | 38.1±31.5 | 32.7±24.3 |  | 11.4±6.9 | 9.30±5.30 |
| de Luis DA8 [R103] | 119 | 14 |  | 41.2±20.9 | 51.2±26 |  | 11.7±6.2 | 7.60±2.50 |
| Oguri K1 [R105] | 51 | 21 |  | 11.2±6.8 | 14.1±6.4 |  | - | - |
| Oguri K2 [R105] | 42 | 18 |  | 11.5±7.2 | 12.3±6.2 |  | - | - |
| de Luis DA9 [R113] | 129 | 15 |  | 37.1±19.4 | 40.1±18.3 |  | 12.1±6.0 | 11.3±5.0 |
| de Luis DA10 [R113] | 119 | 21 |  | 39.8±11.2 | 32.5±11.2 |  | 11.0±4.1 | 10.8±5.0 |
| de Luis DA11 [R118] | 219 | 31 |  | 89.7±46 | 98.4±46 |  | 31.5±42.2 | 35.9±48.6 |
| de Luis DA12 [R118] | 247 | 34 |  | 97.2±56 | 86.7±65 |  | 41.3±34.0 | 40.1±38.0 |
| Daghestani M [R121] | 289 | 40 |  | 22.0±18.02 | 30.12±21.23 |  | - | - |

*ADRB3*: beta3-adrenergic receptor gene; M: mean; SD: standard deviation

**Table S3.** Plasma lipids levels by the genotypes of the *ADRB3* Trp64Arg polymorphism. Data are expressed as M±SD.

| **First author, reference** | **Genotype** | |  | **TG, mmol/L** | |  | **TC, mmol/L** | |  | **LDL-C, mmol/L** | |  | **HDL-C, mmol/L** | |
| --- | --- | --- | --- | --- | --- | --- | --- | --- | --- | --- | --- | --- | --- | --- |
|  | **TT** | **TC+CC** |  | **TT** | **TC+CC** |  | **TT** | **TC+CC** |  | **TT** | **TC+CC** |  | **TT** | **TC+CC** |
| Elbein SC [R1] | 68 | 25 |  | 1.89±1.36 | 1.88±1.10 |  | - | - |  | - | - |  | - | - |
| Urhammer SA [R2] | 331 | 49 |  | 1.1±0.7 | 1.04±0.43 |  | 4.5±0.9 | 4.57±0.82 |  | 2.8±0.8 | 2.87±0.74 |  | 1.2±0.3 | 1.18±0.21 |
| Higashi K [R3] | 49 | 34 |  | 1.71±0.64 | 1.74±0.63 |  | 5.17±0.77 | 5.2±0.72 |  | 3.26±0.72 | 3.29±0.78 |  | 1.14±0.24 | 1.13±0.26 |
| Fujisawa T [R4] | 68 | 33 |  | - | - |  | 5.15±0.83 | 5.45±0.82 |  | - | - |  | - | - |
| Sakane N1 [R5] | 77 | 54 |  | 1.4±0.8 | 1.42±0.64 |  | 4.9±0.7 | 5.46±1.17 |  | - | - |  | 1.4±0.3 | 1.28±0.29 |
| Sakane N2 [R5] | 174 | 82 |  | 0.9±0.3 | 0.89±0.39 |  | 4.8±0.9 | 5.01±0.89 |  | - | - |  | 1.7±0.3 | 1.79±0.3 |
| Yuan XH1 [R6] | 520 | 297 |  | 1.6±1.14 | 1.61±1.07 |  | 5.25±0.85 | 5.30±0.96 |  | - | - |  | 1.46±0.41 | 1.50±0.41 |
| Yuan XH2 [R6] | 187 | 118 |  | 1.13±0.87 | 1±0.4 |  | 5.4±0.9 | 5.31±0.9 |  | - | - |  | 1.73±0.43 | 1.7±0.41 |
| Sakane N3 [R7] | 140 | 75 |  | 1.21±0.58 | 1.66±0.86 |  | 5.09±0.93 | 5.12±0.98 |  | - | - |  | 1.55±0.52 | 1.40±0.52 |
| Rissanen J1 [R8] | 94 | 16 |  | 2.48±1.65 | 2.05±1.44 |  | 6.64 ±1.45 | 6.41±2.16 |  | - | - |  | 1.28±0.39 | 1.37±0.48 |
| Rissanen J2 [R8] | 159 | 24 |  | 2.10±1.51 | 1.94±1.08 |  | 5.71 ±1.01 | 5.80±0.98 |  | - | - |  | 1.15 ±0.25 | 1.19±0.29 |
| Rissanen J3 [R8] | 71 | 11 |  | 1.53±1.18 | 1.40±0.56 |  | 5.96 ±1.10 | 6.12±1.33 |  | - | - |  | 1.28 ±0.25 | 1.26±0.23 |
| ARII K1 [R9] | 26 | 12 |  | 1.94±1.52 | 1.52±1.1 |  | 5.28±0.85 | 5.44±1.48 |  | - | - |  | 1.24±0.39 | 1.29±0.56 |
| ARII K2 [R9] | 13 | 13 |  | 1.36±0.63 | 1.36±0.53 |  | 5.17±0.59 | 5.15±0.98 |  | - | - |  | 1.32±0.44 | 1.45±0.31 |
| ARII K3 [R9] | 85 | 33 |  | 1.34±0.8 | 1.57±1.03 |  | 5.07±0.88 | 5.01±0.84 |  | - | - |  | 1.4±0.31 | 1.36±0.35 |
| ARII K4 [R9] | 67 | 42 |  | 1.01±0.47 | 1.2±0.68 |  | 5.38±0.91 | 5.37±1.06 |  | - | - |  | 1.55±0.34 | 1.41±0.34 |
| Sakane N4 [R10] | 208 | 120 |  | 1.19±0.58 | 1.6±0.8 |  | 5.11±0.95 | 5.19±0.93 |  | - | - |  | 1.58±0.53 | 1.43±0.53 |
| Buettner R1 [R11] | 492 | 67 |  | - | - |  | 5.36±1.19 | 5.76±1.31 |  | - | - |  | 1.33±0.36 | 1.39±0.38 |
| Buettner R2 [R11] | 620 | 80 |  | - | - |  | 5.67±0.94 | 5.57±1.14 |  | - | - |  | 1.7±0.56 | 1.67±0.46 |
| Sun L1 [R12] | 125 | 99 |  | 1.56±1.26 | 1.38±0.83 |  | 5.20±0.79 | 4.97±0.88 |  | 3.19±0.70 | 2.95±0.79 |  | 1.30±0.37 | 1.38±0.36 |
| Sun L2 [R12] | 324 | 198 |  | 1.24±0.72 | 1.22±0.56 |  | 5.26±0.82 | 5.28±0.85 |  | 3.29±0.72 | 3.35±0.78 |  | 1.40±0.31 | 1.38±0.31 |
| Sun L3 [R12] | 121 | 57 |  | 1.99±1.79 | 1.77±1.14 |  | 5.5±1.2 | 5.33±1.08 |  | 3.07±1.16 | 3.16±1.03 |  | 1.47±0.47 | 1.4±0.40 |
| Sun L4 [R12] | 127 | 62 |  | 1.37±0.89 | 1.58±0.80 |  | 5.85±0.83 | 6.03±0.94 |  | 3.51±0.89 | 3.66±0.91 |  | 1.69±0.54 | 1.73±0.48 |
| Esterbauer H [R13] | 133 | 20 |  | 4±4.27 | 4.14±5.49 |  | 5.09±1.03 | 5.25±1.11 |  | - | - |  | 0.88±0.36 | 0.87±0.28 |
| Shima Y [R14] | 176 | 85 |  | 1.27±0.84 | 1.31±0.89 |  | 5.62±0.91 | 5.42±0.81 |  | - | - |  | 1.48±0.36 | 1.42±0.33 |
| Pulkkinen A1 [R15] | 156 | 29 |  | 1.89±0.87 | 1.64±0.65 |  | 6.02± 1 | 5.7±0.81 |  | - | - |  | 1.24 ±0.25 | 1.22±0.22 |
| Pulkkinen A2 [R15] | 103 | 16 |  | 2.54±2.64 | 2.93±1.48 |  | 6.01±1.32 | 6.22±1.36 |  | - | - |  | 1.10±0.3 | 1.09±0.2 |
| Ghosh S [R16] | 1404 | 278 |  | 2.02±1.79 | 2.2±1.94 |  | 5.58±1.19 | 5.69±1.29 |  | - | - |  | 1.18 ±0.33 | 1.14±0.33 |
| Kawamura T [R17] | 101 | 51 |  | 1.79±0.93 | 2.17±1 |  | 5.67±0.99 | 5.9±0.94 |  | - | - |  | 1.37±0.48 | 1.26±0.37 |
| Tonolo G1 [R18] | 184 | 29 |  | 1.4±0.7 | 1.6±0.3 |  | 5.9±1.3 | 5.8±1.6 |  | 3.7±0.9 | 3.7±1.3 |  | 1.5±0.5 | 1.3±0.5 |
| Tonolo G2 [R18] | 262 | 19 |  | 1.4±0.9 | 1.8±0.9 |  | 5.9±1.2 | 5.4±1.1 |  | 3.6±1.0 | 3.3±0.9 |  | 1.5±0.6 | 1.2±0.3 |
| Hayakawa T [R19] | 139 | 75 |  | 1.64±0.60 | 1.76±0.67 |  | 5.42±0.97 | 5.32±0.91 |  | - | - |  | 1.34±0.33 | 1.32±0.35 |
| Sheu WH1 [R20] | 111 | 26 |  | 2.0±2.11 | 1.9±1.02 |  | 5.1±1.05 | 5.0±1.02 |  | 3.3 ±1.05 | 3.3±1.02 |  | 1.0±0 | 0.9±0.51 |
| Sheu WH2 [R20] | 139 | 49 |  | 1.2±1.18 | 1.1±0.7 |  | 4.8±1.18 | 4.6±1.40 |  | 2.9±1.18 | 2.6±1.40 |  | 1.2±0 | 1.2±0.7 |
| Festa A [R21] | 149 | 30 |  | 2.16±0.85 | 2.31±1.04 |  | 6.13±1.22 | 6.56±1.75 |  | - | - |  | - | - |
| Kikuchi T1 [R22] | 45 | 27 |  | 0.84±0.35 | 1.08±0.52 |  | 4.7±0.76 | 4.82±0.78 |  | - | - |  | 1.52±0.31 | 1.48±0.25 |
| Kikuchi T2 [R22] | 83 | 56 |  | 1.14±0.55 | 1.19±0.72 |  | 4.81±0.72 | 4.81±0.69 |  | - | - |  | 1.43±0.28 | 1.42±0.29 |
| Kikuchi T3 [R22] | 23 | 25 |  | 1.16±0.7 | 1.08±0.49 |  | 4.66±0.5 | 4.53±0.73 |  | - | - |  | 1.35±0.25 | 1.35±0.23 |
| Bao AM1 [R23] | 77 | 47 |  | 1.81±1.47 | 1.86±1.99 |  | 5.18±0.98 | 5.01±1.43 |  | - | - |  | 1.30±0.33 | 1.18±0.51 |
| Bao AM2 [R23] | 96 | 42 |  | 1.33± 0.75 | 1.17±0.62 |  | 4.61±0.86 | 4.72±0.99 |  | - | - |  | 1.44±0.40 | 1.49±0.36 |
| Sun LM1 [R24] | 393 | 173 |  | 1.4±1.0 | 1.41±0.7 |  | 5.2±0.9 | 4.99±0.89 |  | 2.4±3.7 | 2.4±0.81 |  | 1.19±0.27 | 1.15±0.27 |
| Sun LM2 [R24] | 114 | 71 |  | 1.7±1.2 | 1.72±0.97 |  | 5.8±1.1 | 5.64±1.17 |  | 3.1±1.0 | 2.98±1.02 |  | 1.30±0.32 | 1.28±0.28 |
| Sun LM3 [R24] | 247 | 157 |  | 1.6±1.3 | 1.36±0.45 |  | 5.2 ±1.0 | 5.02±0.85 |  | 3.2±0.7 | 2.96±0.8 |  | 1.30±0.37 | 1.39±0.36 |
| Sun LM4 [R24] | 455 | 282 |  | 1.2±0.7 | 1.24±0.6 |  | 5.3±0.8 | 5.3±0.84 |  | 3.3±0.7 | 3.32±0.82 |  | 1.40±0.31 | 1.37±0.32 |
| Thomas GN [R25] | 612 | 190 |  | - | - |  | 5.6 ±1.4 | 5.5±1.5 |  | 3.5±1.2 | 3.4±1.2 |  | - | - |
| Strazzullo P [R26] | 869 | 110 |  | 1.7±0.9 | 2.0±1.5 |  | 5.7±1.0 | 5.7±1.1 |  | - | - |  | - | - |
| Urhammer SA [R27] | 184 | 29 |  | 1.1±0.8 | 1.1±0.5 |  | 4.5±0.9 | 4.5±0.7 |  | 2.9±0.8 | 2.8±0.7 |  | 1.2±0.3 | 1.2±0.2 |
| Benecke H [R28] | 157 | 24 |  | 1.84±1.01 | 1.90±0.74 |  | 5.25±1.11 | 5.47±0.93 |  | - | - |  | - | - |
| Pamies-Andreu E [R29] | 71 | 16 |  | 1.83±1.23 | 1.75±0.99 |  | 5.72±1.47 | 5.84±1.16 |  | 3.59 ±1.22 | 3.80±1.03 |  | 1.24±0.36 | 1.22±0.39 |
| Endo K [R30] | 339 | 214 |  | 0.88±0.49 | 0.91±0.43 |  | 4.38±0.63 | 4.43±0.66 |  | 2.58±0.56 | 2.63±0.58 |  | 1.40±0.26 | 1.39±0.31 |
| Corella D1 [R32] | 409 | 33 |  | 1.55 ±1.13 | 2.08±1.72 |  | 5.28±1.06 | 5.85±1.45 |  | 3.46±0.85 | 3.76±1.10 |  | 1.11±0.25 | 1.12±0.27 |
| Corella D2 [R32] | 472 | 65 |  | 0.89±0.47 | 0.87±0.51 |  | 5.01±0.9 | 4.90±0.89 |  | 3.17±0.79 | 3.07±0.84 |  | 1.42±0.27 | 1.40±0.26 |
| Ishii T1 [R33] | 137 | 59 |  | 0.96±0.7 | 1.02±0.43 |  | 4.45±0.8 | 4.5±0.76 |  | 2.74±0.65 | 2.69±0.69 |  | 1.37±0.26 | 1.39±0.34 |
| Ishii T2 [R33] | 125 | 61 |  | 1.51±1.04 | 1.69±1.65 |  | 5.33±0.8 | 5.18±0.74 |  | 3.21±0.75 | 3.07±0.76 |  | 1.42±0.41 | 1.34±0.36 |
| Ishii T3 [R33] | 89 | 33 |  | 1.81±1.08 | 1.71±0.79 |  | 5.12±0.72 | 5.20±0.85 |  | 3.18±0.65 | 3.47±0.75 |  | 1.29±0.36 | 1.11±0.21 |
| Chen Y1 [R34] | 91 | 35 |  | 1.70±0.66 | 1.88±0.68 |  | 4.18±0.94 | 4.32±0.80 |  | - | - |  | 1.35±0.43 | 1.26±0.40 |
| Chen Y2 [R34] | 91 | 36 |  | 1.70±1.15 | 1.65±1.22 |  | 3.81±0.88 | 4.00±0.95 |  | - | - |  | 1.50±0.44 | 1.41±0.28 |
| Wang XL [R35] | 209 | 102 |  | 0.87±0.43 | 0.86±0.39 |  | 4.16±0.72 | 4.17±0.68 |  | 2.19±0.58 | 2.19±0.58 |  | 1.46±0.29 | 1.48±0.29 |
| Chen Y3[R36] | 41 | 32 |  | 1.62±0.49 | 1.92±0.72 |  | 4.11±0.88 | 4.29±0.8 |  | - | - |  | 1.20±0.25 | 1.32±0.42 |
| Chen Y4 [R36] | 64 | 17 |  | 1.74±0.75 | 1.87±0.61 |  | 4.19±1.01 | 4.54±0.64 |  | - | - |  | 1.43±0.49 | 1.29±0.45 |
| Carlsson M [R37] | 64 | 64 |  | 1.3±0.6 | 1.8±1.0 |  | 5.8±1.2 | 6.3±1.2 |  | - | - |  | 1.5±0.4 | 1.40±0.5 |
| Oizumi T [R38] | 1155 | 530 |  | 1.34 ±1.15 | 1.29±0.91 |  | 5.30±0.91 | 5.30±0.93 |  | - | - |  | 1.49 ±0.37 | 1.5±0.40 |
| Manraj M [R39] | 225 | 82 |  | - | - |  | - | - |  | - | - |  | 0.93±0.24 | 0.93±0.24 |
| Yamauchi T [R40] | 46 | 29 |  | 1.25±0.74 | 1.43±0.92 |  | - | - |  | - | - |  | 1.23±0.4 | 1.14±0.37 |
| Wang GY [R41] | 73 | 36 |  | 1.78±1.20 | 1.62±0.70 |  | 5.30 ±1.03 | 5.32±0.96 |  | - | - |  | 1.20±0.35 | 1.22±0.24 |
| Santos JL [R42] | 114 | 38 |  | 1.69±0.93 | 1.55±0.94 |  | 5.01±1.26 | 5.07±1.27 |  | - | - |  | 1.23±0.52 | 1.08±0.35 |
| Matsushita H1 [R43] | 72 | 27 |  | 1.02±0.81 | 1.03±0.66 |  | 4.78±0.67 | 5.00±0.81 |  | 2.63±0.58 | 2.85±0.69 |  | 1.68±0.38 | 1.67±0.41 |
| Matsushita H2 [R43] | 123 | 59 |  | 1.31±0.69 | 1.16±0.53 |  | 5.30±0.91 | 5.21±0.70 |  | 3.14±0.89 | 3.10±0.62 |  | 1.56±0.41 | 1.58±0.32 |
| Matsushita Y1 [R44] | 206 | 122 |  | - | - |  | 5.18±0.9 | 5.17±0.91 |  | - | - |  | 1.44±0.38 | 1.42±0.39 |
| Matsushita Y2 [R44] | 263 | 131 |  | - | - |  | 5.51±0.89 | 5.57±0.89 |  | - | - |  | 1.63±0.38 | 1.61±0.38 |
| Zhu LY1 [R45] | 119 | 56 |  | 1.11±0.35 | 1.11±0.30 |  | 4.89 ±0.73 | 5.14±0.70 |  | 2.83±0.60 | 3.03±0.64 |  | 1.39±0.13 | 1.41±0.10 |
| Zhu LY2 [R45] | 152 | 82 |  | 1.34±0.40 | 1.37±0.41 |  | 5.05 ±0.69 | 4.92±0.75 |  | 2.99±0.64 | 2.90±0.73 |  | 1.39 ±0.14 | 1.38±0.13 |
| Zhu LY3 [R45] | 97 | 55 |  | 1.35±0.43 | 1.47±0.46 |  | 4.98 ±0.51 | 5.17±0.53 |  | 3.01±0.56 | 3.01±0.52 |  | 1.39±0.12 | 1.42±0.08 |
| Tan N1 [R46] | 108 | 21 |  | 1.6±0.5 | 1.7±0.7 |  | 5.4±0.5 | 5.6±0.9 |  | 3.1±0.8 | 3.2±0.7 |  | 1.1±0.3 | 0.9±0.2 |
| Tan N2 [R46] | 99 | 20 |  | 1.5±0.7 | 1.4±1.0 |  | 4.8±0.8 | 4.9±0.4 |  | 2.7±0.3 | 2.8±0.4 |  | 1.2±0.7 | 1.3±0.3 |
| Shiwaku K [R47] | 49 | 27 |  | 1.52±0.82 | 1.17±0.59 |  | 5.35±0.98 | 5.33±0.78 |  | 3.26±0.91 | 3.36±0.75 |  | 1.37±0.31 | 1.42±0.34 |
| Okumura K [R48] | 97 | 39 |  | 1.46±0.94 | 1.82±1.39 |  | 5.34±0.95 | 5.5±0.93 |  | 3.25± 0.95 | 3.35±0.96 |  | 1.41±0.36 | 1.32±0.37 |
| Arashiro R1 [R49] | 34 | 23 |  | 0.73±0.33 | 0.91±0.65 |  | 4.78±0.6 | 4.68±0.87 |  | 2.82±0.6 | 2.87±0.87 |  | 1.6±0.3 | 1.42±0.25 |
| Arashiro R2 [R49] | 31 | 17 |  | 0.91±0.5 | 0.84±0.37 |  | 4.63±1.01 | 4.81±0.75 |  | 2.82± 0.86 | 3.08±0.75 |  | 1.45±0.29 | 1.37±0.32 |
| Kim OY [R50] | 73 | 29 |  | 1.91±0.85 | 2.24±1.62 |  | 5.34±0.94 | 5.56±1.02 |  | 3.29±0.77 | 3.58±1.13 |  | 1.18±0.26 | 1.20±0.32 |
| Gao CR1 [R51] | 77 | 27 |  | 1.31±0.83 | 1.61±0.94 |  | 4.64 ±1.15 | 4.76±1.11 |  | 2.76±0.99 | 2.58±0.82 |  | 1.06±0.39 | 1.08±0.35 |
| Gao CR2 [R51] | 72 | 20 |  | 1.50±0.78 | 2.12±1.65 |  | 4.82 ±1.14 | 4.97±1.35 |  | 3.35±3.35 | 2.62±1.09 |  | 1.17±0.36 | 1.16±0.34 |
| Ramis JM1 [R52] | 30 | 5 |  | 1.67±0.59 | 1.31±0.47 |  | 6.11 ±0.83 | 6.52±1.51 |  | 4.18±0.74 | 4.78±1.50 |  | 1.17±0.27 | 1.14±0.28 |
| Ramis JM2 [R52] | 43 | 7 |  | 1.15±0.53 | 1.25±0.39 |  | 5.28 ±1.05 | 5.89±0.71 |  | 3.44±0.91 | 3.89±0.71 |  | 1.32±0.27 | 1.43±0.27 |
| Ramis JM3 [R52] | 99 | 13 |  | 1.45±0.96 | 1.26±1.05 |  | 5.70 ±1.00 | 5.19±0.94 |  | 3.75±0.89 | 3.30±0.60 |  | 1.29±0.33 | 1.32±0.32 |
| Ramis JM4 [R52] | 103 | 10 |  | 1.04±0.53 | 1.01±0.62 |  | 5.61±0.98 | 5.42±0.77 |  | 3.59±0.83 | 3.38±0.62 |  | 1.54±0.35 | 1.58±0.45 |
| Erhardt E [R53] | 35 | 35 |  | 1.4±0.8 | 1.4±0.6 |  | 4.6±0.8 | 4.3±1.1 |  | - | - |  | 1.2±0.3 | 1.2±0.2 |
| Zuo H [R54] | 125 | 29 |  | 1.90 ±1.50 | 1.99±1.23 |  | 5.28 ±1.02 | 5.04±0.9 |  | - | - |  | 1.20±0.40 | 1.09±0.26 |
| Tan H1 [R55] | 99 | 25 |  | 1.8±0.6 | 1.7±0.7 |  | 5.5±0.9 | 5.7±0.8 |  | - | - |  | - | - |
| Tan H2 [R55] | 47 | 9 |  | 1.2±0.7 | 1.3±0.3 |  | 4.8±0.8 | 4.9±0.4 |  | - | - |  | - | - |
| Miyaki K [R56] | 198 | 97 |  | 1.52±0.97 | 1.44±0.87 |  | 5.45±0.93 | 5.15±1.0 |  | - | - |  | 1.44±0.33 | 1.43±0.42 |
| Perez-Bravo F1 [R57] | 63 | 43 |  | 1.32±0.67 | 1.72±0.97 |  | 4.65±1.15 | 4.79±1.33 |  | - | - |  | 0.86 ±0.4 | 0.86±0.3 |
| Perez-Bravo F2 [R57] | 53 | 29 |  | 1.41±0.55 | 1.27±0.7 |  | 4.57±0.91 | 4.50±1.04 |  | - | - |  | 1.07±0.28 | 1.09±0.36 |
| Lee JS [R58] | 45 | 35 |  | 1.30±0.99 | 1.27±0.68 |  | 5.80±0.96 | 6.16±1.09 |  | 3.52±0.86 | 3.88±1.03 |  | 1.62±0.40 | 1.66±0.44 |
| Kim K1 [R59] | 63 | 32 |  | 1.49±0.8 | 1.34±0.51 |  | 4.73 ±1.02 | 4.81±0.92 |  | 2.64±0.83 | 2.75±0.04 |  | 1.43±1.30 | 1.55 ±0.28 |
| Kim K2 [R59] | 75 | 18 |  | 1.17±0.59 | 0.93±0.34 |  | 4.43±0.66 | 4.22±0.37 |  | 2.39±0.54 | 2.3±0.6 |  | 1.62±0.38 | 1.59 ±0.28 |
| Yuan M [R60] | 228 | 72 |  | - | - |  | 5.3±0.75 | 5.28±0.66 |  | - | - |  | 1.5±0.3 | 1.5±0.33 |
| Tamaki S1 [R61] | 368 | 169 |  | 1.64±1.13 | 1.58±1.06 |  | - | - |  | - | - |  | 1.37±0.37 | 1.42±0.39 |
| Tamaki S2 [R61] | 585 | 294 |  | 1.2±0.7 | 1.23±0.74 |  | - | - |  | - | - |  | 1.6±0.36 | 1.56±0.36 |
| Eshraghi P [R62] | 340 | 61 |  | 1.5±0.8 | 1.8±0.9 |  | 4.8±1 | 5.2±1.1 |  | - | - |  | 1±0.2 | 1±0.3 |
| Klass DM [R63] | 127 | 16 |  | 2.0±1.9 | 1.51±1.16 |  | 5.5 ±1.0 | 5.34±0.98 |  | - | - |  | 1.5±0.5 | 1.47±0.41 |
| Li Q [R64] | 148 | 52 |  | 1.44±0.86 | 1.65±1.33 |  | 4.44 ±0.71 | 4.55±0.88 |  | 2.00±0.54 | 2.12±0.66 |  | 1.55±0.34 | 1.43±0.39 |
| Jiang XY1 [R65] | 43 | 13 |  | 1.88±1.12 | 2.16±1.50 |  | 5.59±1.21 | 5.79±1.41 |  | 3.13±1.01 | 3.48±1.23 |  | 1.38± 0.47 | 1.37±0.28 |
| Jiang XY2 [R65] | 41 | 12 |  | 1.71±0.68 | 1.94±0.76 |  | 5.34±1.65 | 5.98±1.26 |  | 2.97±1.15 | 3.05±0.97 |  | 1.46±0.45 | 1.37±0.53 |
| Chen W1 [R66] | 756 | 135 |  | - | - |  | - | - |  | 3.19±0.85 | 3.15±0.88 |  | - | - |
| Chen W2 [R66] | 289 | 77 |  | - | - |  | - | - |  | 3.04±0.91 | 3.17±1.09 |  | - | - |
| Pierola J [R67] | 316 | 71 |  | 1.83±1.36 | 1.89±1.23 |  | 5.48±1.09 | 5.43±0.98 |  | - | - |  | 1.32±0.34 | 1.27±0.26 |
| Bracale R [R68] | 229 | 13 |  | 1.46±0.70 | 1.48±1.34 |  | - | - |  | 1.29±0.12 | 1.18±0.57 |  | 1.16±0.28 | 1.13±0.28 |
| de Luis DA1 [R69] | 55 | 10 |  | 1.39±0.62 | 1.26±0.50 |  | 5.53±1.19 | 5.12±1.01 |  | 3.19±1.42 | 3.33±1.27 |  | 1.40±0.33 | 1.58±0.85 |
| Ueno T [R70] | 24 | 13 |  | 1.27±0.59 | 1.68±0.61 |  | 5.12±0.96 | 4.82±0.89 |  | - | - |  | 1.38±0.33 | 1.17±0.34 |
| Dunajska K [R71] | 79 | 15 |  | 1.29±0.63 | 1.58±0.72 |  | 6.16±1.07 | 6.11±1.01 |  | 3.84±0.9 | 3.83±0.99 |  | 1.73±0.44 | 1.57±0.31 |
| Yamada K1 [R72] | 78 | 37 |  | 1.98±1.09 | 1.96±1.82 |  | 5.29±0.74 | 5.26±0.69 |  | 3.13±0.79 | 2.99±0.85 |  | 1.25±0.26 | 1.37±0.21 |
| Yamada K2 [R72] | 87 | 30 |  | 1.67±0.84 | 1.67±1.02 |  | 5.65±1.10 | 5.47±0.8 |  | 3.44±0.93 | 3.23±0.73 |  | 1.44±0.27 | 1.46±0.38 |
| Tsuzakia K [R73] | 180 | 97 |  | 1.10±0.49 | 1.23±0.72 |  | 4.79±0.88 | 4.93±0.91 |  | - | - |  | 1.40±0.03 | 1.39±0.38 |
| de Luis DA2 [R74] | 45 | 11 |  | 1.4±0.46 | 1.75±0.86 |  | 5.09±0.80 | 5.74±1.22 |  | 3.07±0.78 | 3.54±0.62 |  | 1.41±0.28 | 1.38±0.31 |
| Kotani K [R75] | 187 | 88 |  | 1.29±1.22 | 1.35±1.32 |  | 5.49±0.90 | 5.73±1.03 |  | 3.15±0.80 | 3.28±1.01 |  | 1.80±0.49 | 1.81±0.52 |
| Chen H [R76] | 690 | 233 |  | 1.66±1.07 | 1.81±1.45 |  | 5.02±1.09 | 5.11±1.07 |  | 2.98±1.84 | 2.97±0.93 |  | 1.35±0.47 | 1.33±0.48 |
| Zhu LY4 [R77] | 171 | 103 |  | 1.46±0.70 | 1.47±0.79 |  | 4.90±0.80 | 4.91±0.82 |  | 2.87±0.76 | 2.90±0.81 |  | 1.46±0.29 | 1.46±0.25 |
| Gjesing AP1 [R78] | 2507 | 396 |  | 1.5±1.4 | 1.53±1.44 |  | 5.6±1.1 | 5.63±1.17 |  | - | - |  | 1.30 ±0.40 | 1.3±0.4 |
| Gjesing AP2 [R78] | 2525 | 394 |  | 1.1±1.3 | 1.2±0.8 |  | 5.4±1.1 | 5.51±1.01 |  | - | - |  | 1.6±0.4 | 1.6±0.4 |
| Nonen S [R79] | 129 | 59 |  | 1.7±1.0 | 1.61±1.27 |  | 5.3±0.9 | 5.09±0.85 |  | 3.1±0.9 | 2.95±0.72 |  | 1.4±0.4 | 1.4±0.39 |
| Hamada T [R80] | 29 | 15 |  | 0.68±0.38 | 0.74±0.24 |  | 4.65±0.78 | 4.84±0.84 |  | 2.53±0.75 | 2.73±0.83 |  | 1.78±0.39 | 1.77±0.36 |
| Dunajska K [R81] | 243 | 41 |  | 1.2±0.5 | 1.36±0.61 |  | 6.38±1.09 | 6.2±1.04 |  | 3.99±1 | 3.86±1.06 |  | 1.85±0.45 | 1.65±0.35 |
| Zafarmand MH [R82] | 1331 | 188 |  | - | - |  | 5.9 ±1 | 6.0±1 |  | 3.9±0.9 | 4.0±1.0 |  | 1.6±0.4 | 1.6±0.4 |
| de Luis DA3 [R83] | 87 | 9 |  | 1.51±0.84 | 1.51±0.54 |  | 5.30±0.93 | 5.72±0.75 |  | 3.18±0.96 | 3.36±1.27 |  | 1.37±0.38 | 1.27±0.48 |
| de Luis DA4 [R83] | 85 | 12 |  | 1.25±0.6 | 1.21±0.7 |  | 5.03±1.14 | 5.21±1.23 |  | 3.06±1.01 | 3.15±0.83 |  | 1.45±0.27 | 1.41±0.47 |
| Zhu LY5 [R84] | 249 | 137 |  | 1.3±0.41 | 1.4±0.43 |  | 5.0±0.63 | 5.1±0.71 |  | 3.0±0.61 | 3.0±0.70 |  | 1.4±0.14 | 1.4±0.11 |
| Yu B [R85] | 1764 | 950 |  | - | - |  | 5.17±0.90 | 5.26±0.88 |  | - | - |  | 1.58±0.4 | 1.58±0.41 |
| Zhang JH [R86] | 115 | 65 |  | 0.85±0.43 | 0.62±0.25 |  | 3.51 ±0.61 | 3.12±0.55 |  | 2.12±0.53 | 1.83±0.53 |  | 0.99±0.09 | 1.01±0.03 |
| Yang M [R87] | 187 | 86 |  | 3.12±3.74 | 4.29±5.27 |  | 4.66± 2.01 | 4.50±2.28 |  | 2.92±1.09 | 3.18±1.52 |  | 1.37± 0.83 | 1.09±0.33 |
| Li HH [R88] | 178 | 77 |  | 3.1±3.7 | 4.3±5.3 |  | 4.7±2.0 | 4.5±2.3 |  | 2.2±1.1 | 3.2±1.5 |  | 1.4±0.83 | 1.1±0.3 |
| Chen YZ1 [R89] | 26 | 15 |  | 1.5±0.7 | 1.1±0.5 |  | 5.00 ±1.20 | 4.50±0.80 |  | 3.40±0.91 | 3.10±0.90 |  | 1.20±0.20 | 1.00±0.30 |
| Chen YZ2 [R89] | 33 | 17 |  | 1.7±1.3 | 1.7±0.9 |  | 4.80 ±1.00 | 4.90±0.70 |  | 3.00±1.20 | 3.10±1.10 |  | 1.20±0.40 | 1.40±0.30 |
| Zhu LY6 [R90] | 294 | 188 |  | 1.32±0.43 | 1.44±0.31 |  | 5.12±0.68 | 5.21±0.73 |  | 2.94±0.63 | 2.95±0.70 |  | 1.41±0.15 | 1.40±0.13 |
| Zhu LY7 [R90] | 158 | 81 |  | 1.12±0.34 | 1.15±0.42 |  | 4.92±0.65 | 5.09±0.70 |  | 2.84±0.65 | 3.02±0.69 |  | 1.39±0.12 | 1.41±0.10 |
| Kim SM1 [R91] | 25 | 8 |  | 1.66±2.26 | 0.79±0.37 |  | 4.17±1.68 | 4.01±0.54 |  | 2.22±1.10 | 2.45±0.42 |  | 1.18±0.22 | 1.20±0.24 |
| Kim SM2 [R91] | 65 | 16 |  | 1.06±0.55 | 0.91±0.34 |  | 4.07±0.64 | 3.97±0.59 |  | 2.27±0.51 | 2.07±0.44 |  | 1.31±0.25 | 1.48±0.32 |
| de Luis DA5 [R92] | 226 | 38 |  | 1.38±0.76 | 1.32±0.62 |  | 4.86±0.96 | 4.8±1 |  | 2.79±1.1 | 2.66±1.11 |  | 1.71±0.94 | 1.64±0.85 |
| Morcillo S [R93] | 603 | 88 |  | 1.11±0.82 | 1.01±0.52 |  | 5.07±1.07 | 5.10±1.13 |  | - | - |  | - | - |
| Peng AN [R94] | 252 | 105 |  | 0.96±0.70 | 1±0.76 |  | 4.13±0.61 | 4.05±0.7 |  | 2.09±0.38 | 2.18±0.46 |  | 1.64±0.23 | 1.56±0.32 |
| Genelhu VA [R95] | 120 | 20 |  | 1.73±0.94 | 1.8±0.93 |  | 5.23 ±1.11 | 5.17±1.07 |  | 3.28±0.93 | 3.06±0.82 |  | 1.16±0.26 | 1.28±0.29 |
| Tsunekawa K [R96] | 142 | 80 |  | 1.16±0.64 | 1.3±0.78 |  | 5.45±0.88 | 5.66±0.84 |  | - | - |  | 1.6±0.41 | 1.55±0.36 |
| Mirrakhimov AE [R97] | 116 | 97 |  | - | - |  | 5.14±1.08 | 4.92±0.9 |  | 3.23±0.92 | 3.12±0.8 |  | 1.12±0.34 | 1.02±0.33 |
| Malik SG1 [R98] | 157 | 21 |  | 1.93±1.15 | 1.82±0.71 |  | - | - |  | - | - |  | 1.22±0.26 | 1.14±0.26 |
| Malik SG2 [R98] | 94 | 13 |  | 1.31±0.70 | 1.29±0.78 |  | - | - |  | - | - |  | 1.45±0.28 | 1.47±0.23 |
| Malik SG3 [R98] | 113 | 15 |  | 1.64±0.69 | 1.41±0.42 |  | - | - |  | - | - |  | 1.24±0.26 | 1.29±0.26 |
| Malik SG4 [R98] | 107 | 11 |  | 1.32 ± 0.59 | 1.16±0.56 |  | - | - |  | - | - |  | 1.47±0.31 | 1.42±0.21 |
| Iwamoto Y [R99] | 265 | 92 |  | 1.67±0.98 | 1.54±0.94 |  | 5.35±0.93 | 5.33±0.83 |  | - | - |  | 1.5±0.44 | 1.58±0.47 |
| Kwon DH [R100] | 33 | 15 |  | 1.32±0.54 | 1.62±0.07 |  | 4.96±0.92 | 5.09±1.4 |  | - | - |  | 1.42±0.24 | 1.21±0.19 |
| Zhang XY [R101] | 143 | 52 |  | 1.60±0.95 | 1.8±1.01 |  | 4.65±1.19 | 5.06±0.88 |  | 2.67±0.74 | 2.71±0.69 |  | - | - |
| de Luis DA6 [R102] | 162 | 50 |  | 1.37±0.62 | 1.40±0.86 |  | 5.36±1.19 | 5.22±0.96 |  | 3.29±1.42 | 3.28±1.97 |  | 1.41±0.33 | 1.43±0.33 |
| de Luis DA7 [R103] | 112 | 15 |  | 1.36±0.56 | 1.43±0.74 |  | 5.37±0.88 | 5.25±1.01 |  | 3.36±0.78 | 3.27±1.06 |  | 1.38±0.28 | 1.26±0.26 |
| de Luis DA8 [R103] | 119 | 14 |  | 1.47±0.6 | 1.21±0.7 |  | 5.37±1.22 | 5.21±1.23 |  | 3.45±1.04 | 3.15±0.83 |  | 1.31±0.32 | 1.41±0.47 |
| Hameed I [R104] | 49 | 151 |  | 1.77±0.59 | 2.58±1.46 |  | 3.94±0.83 | 4.41±1.48 |  | 2.45±0.51 | 2.82±0.69 |  | 1.04±0.19 | 0.98±0.25 |
| Oguri K1 [R105] | 51 | 21 |  | 0.79±0.42 | 1.15±0.43 |  | 4.84±0.75 | 5.21±0.82 |  | 2.68±0.54 | 3.22±2.68 |  | 1.64±0.35 | 1.54±0.36 |
| Oguri K2 [R105] | 42 | 18 |  | 0.87±0.39 | 1.17±0.51 |  | 4.78±0.5 | 5.17±0.67 |  | 2.65±0.57 | 3.12±0.55 |  | 1.57±0.24 | 1.56±0.24 |
| Zhang XL [R106] | 41 | 21 |  | 1.56±0.56 | 1.60±0.68 |  | 4.55±0.79 | 4.78±1.02 |  | 2.76±0.79 | 2.81±0.87 |  | 1.30±0.51 | 1.25±0.38 |
| Kumar S [R107] | 422 | 178 |  | 1.67±0.72 | 1.57±0.64 |  | 3.59±0.96 | 3.41±1.09 |  | 1.94±0.72 | 1.88±0.73 |  | 0.87±0.20 | 0.85±0.23 |
| Brondani LA [R108] | 859 | 156 |  | - | - |  | 5.3±1.3 | 5.4±1.3 |  | 3.30±1.24 | 3.34±1.28 |  | 1.11±0.4 | 1.18±0.4 |
| Iemitsu M [R109] | 590 | 287 |  | 0.93±0.55 | 0.94±0.53 |  | 5.17±0.63 | 5.19±0.84 |  | - | - |  | 1.68±0.63 | 1.72±0.4 |
| Wang T [R110] | 446 | 222 |  | 1.19±0.58 | 1.08±0.59 |  | 5.38±0.88 | 5.46±0.85 |  | 3.1±0.72 | 3.15±0.67 |  | 1.47±0.34 | 1.52±0.32 |
| Yoshihara A [R111] | 206 | 126 |  | 1.12±0.59 | 1.24±0.67 |  | 5.62 ±0.74 | 5.69±0.83 |  | - | - |  | - | - |
| Hui ZG [R112] | 77 | 32 |  | 1.88±0.73 | 2.09±1.12 |  | 5.27±1.22 | 5.32±0.72 |  | 3.14±0.88 | 3.25±0.76 |  | 1.13±0.23 | 0.93±0.22 |
| de Luis DA9 [R113] | 129 | 15 |  | 1.39±0.34 | 1.34±0.23 |  | 5.42±0.69 | 5.21±0.31 |  | 3.33±0.70 | 3.14±0.42 |  | 1.41±0.27 | 1.44±0.24 |
| de Luis DA10 [R113] | 119 | 21 |  | 1.42±0.45 | 1.43±0.44 |  | 5.41±0.78 | 5.41±0.32 |  | 3.33±0.55 | 3.22±0.49 |  | 1.48±0.28 | 1.44±0.27 |
| Chen YH1 [R114] | 184 | 65 |  | 2.40±1.79 | 1.95±1.11 |  | 5.58 ±1.02 | 5.33±0.77 |  | 3.32± 0.91 | 3.19±0.73 |  | 1.16±0.31 | 1.24±0.36 |
| Chen YH2 [R114] | 124 | 45 |  | 1.64±1.05 | 2.05±1.54 |  | 5.36±1.04 | 5.77±1.42 |  | 3.21± 1.01 | 3.42±1.15 |  | 1.41±0.41 | 1.44±0.42 |
| Verdi H [R115] | 108 | 22 |  | 1.27±0.69 | 1.30±0.53 |  | -- | - |  | 2.53±0.64 | 2.51±0.48 |  | 1.13±0.03 | 1.06±0.21 |
| Liu JL [R116] | 1126 | 476 |  | 1.96±1.35 | 1.88 ±1.64 |  | 5.16± 0.99 | 5.08±0.96 |  | 3.43±0.85 | 3.31±0.84 |  | 1.18±0.24 | 1.20±0.25 |
| Dong CM [R117] | 74 | 21 |  | 2.4±0.4 | 2.2±0.6 |  | 5.3±0.6 | 5.6±0.9 |  | 3.5±0.8 | 3.3±0.7 |  | 1.0±0.3 | 0.9±0.2 |
| de Luis DA11 [R118] | 219 | 31 |  | 1.54±0.69 | 1.39±0.53 |  | 5.41±0.96 | 5.35±0.93 |  | 3.31±0.96 | 3.13±0.91 |  | 1.44±0.7 | 1.73±1.29 |
| de Luis DA12 [R118] | 247 | 34 |  | 1.06±0.39 | 1.25±0.7 |  | 5.04±1.03 | 5.25±1.06 |  | 3.15±0.88 | 3.18±1.06 |  | 1.5±0.42 | 1.40±0.35 |
| Jesus IC [R119] | 54 | 18 |  | - | - |  | 3.84±0.61 | 4.15±0.82 |  | 1.99±0.44 | 2.34±0.64 |  | 1.39±0.25 | 1.42±0.28 |
| Milano-Gai GE [R120] | 65 | 18 |  | 1.31±0.68 | 1.62±1.05 |  | 4.12±0.87 | 4.15±0.84 |  | 2.41±0.73 | 2.13±0.54 |  | 1.12±0.19 | 1.12±0.16 |
| Daghestani M [R121] | 289 | 40 |  | 1.02±0.51 | 1.37±0.52 |  | 3.94±0.85 | 4.58±1.07 |  | 2.05±0.85 | 2.49±0.9 |  | 1.19±0.34 | 1.11±0.29 |
| Grygiel-Gorniak B1 [R122] | 158 | 35 |  | 1.16±0.42 | 1.36±0.63 |  | - | - |  | - | - |  | 1.69±0.39 | 1.69±0.39 |
| Grygiel-Gorniak B2 [R122] | 61 | 16 |  | 1.72±0.79 | 1.47±0.62 |  | - | - |  | - | - |  | 1.54±0.36 | 1.66±0.30 |

*ADRB3*: beta3-adrenergic receptor gene; TG: triglycerides; TC: total cholesterol; LDL-C: low-density lipoprotein cholesterol; HDL-C: high-density lipoprotein cholesterol; M: mean; SD: standard deviation

**Table S4.** Meta-regression analysis explore the sources of heterogeneity of plasma triglycerides (TG) levels.

| Heterogeneity factors | Coefficient | SE | z | p | 95 % CI |
| --- | --- | --- | --- | --- | --- |
| Publication year |  |  |  |  |  |
| Univariate | -0.02 | 0.05 | -0.31 | 0.76 | -0.11-0.08 |
| Multivariate | 0.00 | 0.05 | 0.01 | 0.99 | -0.101-0.101 |
| Language |  |  |  |  |  |
| Univariate | 0.02 | 0.05 | 0.44 | 0.66 | -0.07-0.11 |
| Multivariate | 0.01 | 0.05 | 0.16 | 0.87 | -0.09-0.11 |
| Ethnicity |  |  |  |  |  |
| Univariate | -0.02 | 0.04 | -0.54 | 0.59 | -0.09-0.05 |
| Multivariate | -0.02 | 0.04 | -0.52 | 0.61 | -0.09-0.05 |
| Gender |  |  |  |  |  |
| Univariate | 0.02 | 0.03 | 0.90 | 0.37 | -0.03-0.07 |
| Multivariate | 0.01 | 0.03 | 0.28 | 0.78 | -0.05-0.06 |
| Disease status |  |  |  |  |  |
| Univariate | -0.02 | 0.02 | -1.10 | 0.27 | -0.06-0.02 |
| Multivariate | -0.02 | 0.02 | -1.01 | 0.31 | -0.06-0.02 |
| Total sample size |  |  |  |  |  |
| Univariate | -0.08 | 0.04 | -1.99 | 0.05 | -0.16--0.00 |
| Multivariate | -0.07 | 0.04 | -1.72 | 0.09 | -0.16-0.01 |

**Table S5. Meta-regression analysis explore the sources of heterogeneity of plasma high-density lipoprotein cholesterol (HDL-C) levels.**

| Heterogeneity factors | Coefficient | SE | z | p | 95 % CI |
| --- | --- | --- | --- | --- | --- |
| Publication year |  |  |  |  |  |
| Univariate | 0.03 | 0.04 | 0.64 | 0.53 | -0.06-0.11 |
| Multivariate | 0.04 | 0.04 | 0.95 | 0.34 | -0.04-0.13 |
| Language |  |  |  |  |  |
| Univariate | 0.04 | 0.04 | 0.93 | 0.35 | -0.04-0.12 |
| Multivariate | 0.05 | 0.04 | 1.24 | 0.22 | -0.03-0.13 |
| Ethnicity |  |  |  |  |  |
| Univariate | -0.00 | 0.03 | -0.12 | 0.91 | -0.06-0.06 |
| Multivariate | 0.00 | 0.03 | 0.15 | 0.88 | -0.06-0.07 |
| Gender |  |  |  |  |  |
| Univariate | -0.03 | 0.02 | -1.33 | 0.19 | -0.07-0.01 |
| Multivariate | -0.01 | 0.02 | -0.56 | 0.57 | -0.06-0.03 |
| Disease status |  |  |  |  |  |
| Univariate | 0.02 | 0.02 | 1.07 | 0.29 | -0.02-0.05 |
| Multivariate | 0.01 | 0.02 | 0.94 | 0.35 | -0.02-0.05 |
| Total sample size |  |  |  |  |  |
| Univariate | 0.09 | 0.03 | 2.39 | <0.01 | 0.03-0.16 |
| Multivariate | 0.09 | 0.03 | 2.49 | 0.01 | 0.02-0.15 |

**Table S6. Meta-regression analysis explore the sources of heterogeneity of plasma adiponectin levels.**

| Heterogeneity factors | Coefficient | SE | z | p | 95 % CI |
| --- | --- | --- | --- | --- | --- |
| Language |  |  |  |  |  |
| Univariate | 0.65 | 0.33 | 1.96 | 0.08 | -0.09-1.39 |
| Multivariate | 0.01 | 0.20 | 0.06 | 0.95 | -0.44-0.46 |
| Disease status |  |  |  |  |  |
| Univariate | 0.39 | 0.06 | 6.29 | <0.001 | 0.25-0.52 |
| Multivariate | 0.36 | 0.08 | 4.40 | <0.001 | 0.17-0.55 |
| Total sample size |  |  |  |  |  |
| Univariate | 0.39 | 0.28 | 1.39 | 0.20 | -0.24-1.02 |
| Multivariate | 0.16 | 0.16 | 1.02 | 0.34 | -0.21-0.53 |

**Table S7. Meta-regression analysis explore the sources of heterogeneity of plasma leptin levels.**

| Heterogeneity factors | Coefficient | SE | z | p | 95 % CI |
| --- | --- | --- | --- | --- | --- |
| Publication year |  |  |  |  |  |
| Univariate | 0.14 | 0.36 | 0.40 | 0.70 | -0.59-0.88 |
| Multivariate | 0.11 | 0.39 | 0.27 | 0.79 | -0.70-0.91 |
| Ethnicity |  |  |  |  |  |
| Univariate | 0.02 | 0.09 | 0.26 | 0.80 | -0.16-0.20 |
| Multivariate | 0.00 | 0.11 | 0.03 | 0.98 | -0.23-0.23 |
| Gender |  |  |  |  |  |
| Univariate | -0.04 | 0.07 | -0.61 | 0.55 | -0.19-0.10 |
| Multivariate | -0.07 | 0.10 | -0.67 | 0.51 | -0.27-0.14 |
| Disease status |  |  |  |  |  |
| Univariate | -0.03 | 0.10 | -0.33 | 0.74 | -0.24-0.18 |
| Multivariate | -0.02 | 0.12 | -0.15 | 0.88 | -0.26-0.22 |
| Total sample size. |  |  |  |  |  |
| Univariate | -0.03 | 0.14 | -0.21 | 0.84 | -0.31-0.25 |
| Multivariate | -0.10 | 0.18 | -0.59 | 0.56 | -0.47-0.26 |

**Table S8.** Meta-regression analysis explore the sources of heterogeneity of plasma total cholesterol (TC) levels.

| Heterogeneity factors | Coefficient | SE | z | p | 95 % CI |
| --- | --- | --- | --- | --- | --- |
| Publication year |  |  |  |  |  |
| Univariate | 0.01 | 0.04 | 0.23 | 0.82 | -0.07-0.09 |
| Multivariate | 0.01 | 0.04 | 0.32 | 0.75 | -0.07-0.10 |
| Language |  |  |  |  |  |
| Univariate | 0.01 | 0.04 | 0.37 | 0.71 | -0.06-0.09 |
| Multivariate | 0.01 | 0.04 | 0.34 | 0.74 | -0.07-0.10 |
| Ethnicity |  |  |  |  |  |
| Univariate | -0.03 | 0.03 | -0.87 | 0.38 | -0.09-0.03 |
| Multivariate | -0.03 | 0.03 | -0.90 | 0.37 | -0.09-0.03 |
| Gender |  |  |  |  |  |
| Univariate | 0.02 | 0.02 | 0.78 | 0.44 | -0.03-0.06 |
| Multivariate | 0.01 | 0.02 | 0.66 | 0.51 | -0.03-0.06 |
| Disease status |  |  |  |  |  |
| Univariate | 0.00 | 0.01 | 0.19 | 0.85 | -0.03-0.03 |
| Multivariate | 0.00 | 0.02 | 0.21 | 0.84 | -0.03-0.03 |
| Total sample size. |  |  |  |  |  |
| Univariate | -0.02 | 0.03 | -0.75 | 0.45 | -0.09-0.04 |
| Multivariate | -0.02 | 0.03 | -0.60 | 0.55 | -0.09-0.05 |

**Table S9.** Meta-regression analysis explore the sources of heterogeneity for circulating low-density lipoprotein cholesterol (LDL-C) levels.

| Heterogeneity factors | Coefficient | SE | z | p | 95 % CI |
| --- | --- | --- | --- | --- | --- |
| Publication year |  |  |  |  |  |
| Univariate | 0.01 | 0.04 | 0.23 | 0.82 | -0.07-0.09 |
| Multivariate | 0.07 | 0.08 | 0.84 | 0.40 | -0.09-0.22 |
| Language |  |  |  |  |  |
| Univariate | 0.02 | 0.05 | 0.36 | 0.72 | -0.09-0.13 |
| Multivariate | 0.02 | 0.06 | 0.43 | 0.67 | -0.09-0.14 |
| Ethnicity |  |  |  |  |  |
| Univariate | 0.07 | 0.04 | 1.56 | 0.12 | -0.02-0.15 |
| Multivariate | 0.06 | 0.04 | 1.26 | 0.21 | -0.03-0.14 |
| Gender |  |  |  |  |  |
| Univariate | 0.03 | 0.03 | 1.06 | 0.29 | -0.03-0.10 |
| Multivariate | 0.02 | 0.03 | 0.46 | 0.65 | -0.05-0.08 |
| Disease status |  |  |  |  |  |
| Univariate | -0.04 | 0.02 | -1.64 | 0.10 | -0.08-0.01 |
| Multivariate | -0.02 | 0.02 | -1.07 | 0.29 | -0.07-0.02 |
| Total sample size. |  |  |  |  |  |
| Univariate | -0.04 | 0.05 | -0.91 | 0.37 | -0.14-0.05 |
| Multivariate | -0.03 | 0.05 | -0.63 | 0.53 | -0.13-0.07 |

**Supplementary References**

[R1] Elbein SC, Hoffman M, Barrett K, et al. Role of the beta 3-adrenergic receptor locus in obesity and noninsulin-dependent diabetes among members of Caucasian families with a diabetic sibling pair. J Clin Endocrinol Metab 1996;81:4422-7.

[R2] Urhammer SA, Clausen JO, Hansen T, Pedersen O. Insulin sensitivity and body weight changes in young white carriers of the codon 64 amino acid polymorphism of the beta 3-adrenergic receptor gene. Diabetes 1996;45:1115-20.

[R3] Higashi K, Ishikawa T, Ito T, Yonemura A, Shige H, Nakamura H. Association of a genetic variation in the beta 3-adrenergic receptor gene with coronary heart disease among Japanese. Biochem Biophys Res Commun 1997;232:728-30.

[R4] Fujisawa T, Ikegami H, Yamato E, et al. Trp64Arg mutation of beta3-adrenergic receptor in essential hypertension: insulin resistance and the adrenergic system. Am J Hypertens 1997;10:101-5.

[R5] Sakane N, Yoshida T, Umekawa T, Kondo M, Sakai Y, Takahashi T. Beta 3-adrenergic-receptor polymorphism: a genetic marker for visceral fat obesity and the insulin resistance syndrome. Diabetologia 1997;40:200-4.

[R6] Yuan X, Yamada K, Koyama K, et al. Beta 3-adrenergic receptor gene polymorphism is not a major genetic determinant of obesity and diabetes in Japanese general population. Diabetes Res Clin Pract 1997;37:1-7.

[R7] Sakane N, Yoshida T, Yoshioka K, et al. Beta 3-adrenoreceptor gene polymorphism: a newly identified risk factor for proliferative retinopathy in NIDDM patients. Diabetes 1997;46:1633-6.

[R8] Rissanen J, Kuopusjärvi J, Pihlajamäki J, et al. The Trp64Arg polymorphism of the beta 3-Adrenergic receptor gene. Lack of association with NIDDM and features of insulin resistance syndrome. Diabetes Care 1997;20:1319-23.

[R9] Arii K, Suehiro T, Yamamoto M, et al. Trp64Arg mutation of beta 3-adrenergic receptor and insulin sensitivity in subjects with glucose intolerance. Intern Med 1997;36:603-6.

[R10] Sakane N, Yoshida T, Yoshioka K, et al. Trp64Arg mutation of beta3-adrenoceptor gene is associated with diabetic nephropathy in Type II diabetes mellitus. Diabetologia 1998;41:1533-4.

[R11] Büettner R, Schäffler A, Arndt H, et al. The Trp64Arg polymorphism of the beta 3-adrenergic receptor gene is not associated with obesity or type 2 diabetes mellitus in a large population-based Caucasian cohort. J Clin Endocrinol Metab 1998;83:2892-7.

[R12] Sun L, Ishibashi S, Osuga J, et al. Clinical features associated with the homozygous Trp64Arg mutation of the beta3-adrenergic receptor: no evidence for its association with obesity in Japanese. Arterioscler Thromb Vasc Biol 1998;18:941-6.

[R13] Esterbauer H, Oberkofler H, Liu YM, et al. Uncoupling protein-1 mRNA expression in obese human subjects: the role of sequence variations at the uncoupling protein-1 gene locus. J Lipid Res 1998;39:834-44.

[R14] Shima Y, Tsukada T, Nakanishi K, Ohta H. Association of the Trp64Arg mutation of the beta3-adrenergic receptor with fatty liver and mild glucose intolerance in Japanese subjects. Clin Chim Acta 1998;274:167-76.

[R15] Pulkkinen A, Kareinen A, Saarinen L, Heikkinen S, Lehto S, Laakso M. The codon 64 polymorphism of the beta3-adrenergic receptor gene is not associated with coronary heart disease or insulin resistance in nondiabetic subjects and non-insulin-dependent diabetic patients. Metabolism 1999;48:853-6.

[R16] Ghosh S, Langefeld CD, Ally D, et al. The W64R variant of the beta3-adrenergic receptor is not associated with type II diabetes or obesity in a large Finnish sample. Diabetologia 1999;42:238-44.

[R17] Kawamura T, Egusa G, Okubo M, Imazu M, Yamakido M. Association of beta3-adrenergic receptor gene polymorphism with insulin resistance in Japanese-American men. Metabolism 1999;48:1367-70.

[R18] Tonolo G, Melis MG, Secchi G, et al. Association of Trp64Arg beta 3-adrenergic-receptor gene polymorphism with essential hypertension in the Sardinian population. J Hypertens 1999;17:33-8.

[R19] Hayakawa T, Nagai Y, Taniguchi M, et al. Phenotypic characterization of the beta3-adrenergic receptor mutation and the uncoupling protein 1 polymorphism in Japanese men. Metabolism 1999;48:636-40.

[R20] Sheu WH, Lee WJ, Yao YE, Jeng CY, Young MM, Chen YT. Lack of association between genetic variation in the beta3-adrenergic receptor gene and insulin resistance in patients with coronary heart disease. Metabolism 1999;48:651-4.

[R21] Festa A, Krugluger W, Shnawa N, Hopmeier P, Haffner SM, Schernthaner G. Trp64Arg polymorphism of the beta3-adrenergic receptor gene in pregnancy: association with mild gestational diabetes mellitus. J Clin Endocrinol Metab 1999;84:1695-9.

[R22] Kikuchi T, Hashimoto N, Kawasaki T, Okugawa T, Uchiyama M. Association Between a Polymorphism in the β 3-adrenergic Receptor Gene and Childhood Obesity. Aeta Medico et Biologica 2000;48:135-9.

[R23] Bao AM, Xiao SX, Wang CJ, Yang MG. Phenotypic characteristics of the Trp64Arg polymorphism in the beta 3-adrenergic receptor gene in type 2 diabetes and normal subjects. Chinese Journal of Diabetes 2000;8:13-6.

[R24] Sun LM, Yoko Iizuka, Shun Ishibashi et al. Investigation and comparison of the β3-adrenergic receptor gene Trp64Arg mutation in the Chinese and Japanese. Nat l Med J China 2000;80:107-10.

[R25] Thomas GN, Tomlinson B, Chan JC, Young RP, Critchley JA. The Trp64Arg polymorphism of the beta3-adrenergic receptor gene and obesity in Chinese subjects with components of the metabolic syndrome. Int J Obes Relat Metab Disord 2000;24:545-51.

[R26] Strazzullo P, Iacone R, Siani A, et al. Relationship of the Trp64Arg polymorphism of the beta3-adrenoceptor gene to central adiposity and high blood pressure: interaction with age. Cross-sectional and longitudinal findings of the Olivetti Prospective Heart Study. J Hypertens 2001;19:399-406.

[R27] Urhammer SA, Hansen T, Borch-Johnsen K, Pedersen O. Studies of the synergistic effect of the Trp/Arg64 polymorphism of the beta3-adrenergic receptor gene and the -3826 A-->G variant of the uncoupling protein-1 gene on features of obesity and insulin resistance in a population-based sample of 379 young Danish subjects. J Clin Endocrinol Metab 2000;85:3151-4.

[R28] Benecke H, Topak H, von zur Mühlen A, Schuppert F. A study on the genetics of obesity: influence of polymorphisms of the beta-3-adrenergic receptor and insulin receptor substrate 1 in relation to weight loss, waist to hip ratio and frequencies of common cardiovascular risk factors. Exp Clin Endocrinol Diabetes 2000;108:86-92.

[R29] Pamies-Andreu E, García-Lozano R, Palmero-Palmero C, et al. Genetic variation in the beta-3-adrenergic receptor in essential hypertension. Life Sci 2000;67:391-7.

[R30] Endo K, Yanagi H, Hirano C, Hamaguchi H, Tsuchiya S, Tomura S. Association of Trp64Arg polymorphism of the beta3-adrenergic receptor gene and no association of Gln223Arg polymorphism of the leptin receptor gene in Japanese schoolchildren with obesity. Int J Obes Relat Metab Disord 2000;24:443-9.

[R31] Lowe WL Jr, Rotimi CN, Luke A, et al. The beta 3-adrenergic receptor gene and obesity in a population sample of African Americans. Int J Obes Relat Metab Disord 2001;25:54-60.

[R32] Corella D, Guillén M, Portolés O, et al. Gender specific associations of the Trp64Arg mutation in the beta3-adrenergic receptor gene with obesity-related phenotypes in a Mediterranean population: interaction with a common lipoprotein lipase gene variation. J Intern Med 2001;250:348-60.

[R33] Ishii T, Hirose H, Kawai T, et al. Effects of intestinal fatty acid-binding protein gene Ala54Thr polymorphism and beta3-adrenergic receptor gene Trp64Arg polymorphism on insulin resistance and fasting plasma glucose in young to older Japanese men. Metabolism 2001;50:1301-7.

[R34] Chen Y, Zhou L, Xu YC, et al. Study on Association of Mutation of the β3 -adrenergic-receptor Gene with NIDDM. China Public Health 2001; 17: 586-8.

[R35] Xinli W, Xiaomei T, Meihua P, Song L. Association of a mutation in the beta3-adrenergic receptor gene with obesity and response to dietary intervention in Chinese children. Acta Paediatr 2001;90:1233-7.

[R36] Chen Y, Xu YC, Zhou L, Shen HB, YU RB, Niu JY. Association of β3-adrenergic receptor gene with obesity in patient with type 2 diabetes mellitus. Chinese Journal of Preventive Medicine 2001;35(5):333-5.

[R37] Carlsson M, Orho-Melander M, Hedenbro J, Groop LC. Common variants in the beta2-(Gln27Glu) and beta3-(Trp64Arg)--adrenoceptor genes are associated with elevated serum NEFA concentrations and type II diabetes. Diabetologia 2001;44:629-36.

[R38] Oizumi T, Daimon M, Saitoh T, et al. Genotype Arg/Arg, but not Trp/Arg, of the Trp64Arg polymorphism of the beta(3)-adrenergic receptor is associated with type 2 diabetes and obesity in a large Japanese sample. Diabetes Care 2001;24:1579-83.

[R39] Manraj M, Francke S, Hébé A, Ramjuttun US, Froguel P. Genetic and environmental nature of the insulin resistance syndrome in Indo-Mauritian subjects with premature coronary heart disease: contribution of beta3-adrenoreceptor gene polymorphism and beta blockers on triglyceride and HDL concentrations. Diabetologia 2001;44:115-22.

[R40] Yamauchi T, Kuno T, Takada H, et al. The impact of Trp64Arg mutation in the beta3-adrenergic receptor gene on haemodialysis patients. Nephrol Dial Transplant 2001;16:641-2.

[R41] Wang GY, Li QF, Niu TH, Chen CZ, Xu XP. Association of GYS1 and β3-AR gene with postprandial hyperglycemia and serum uric acid in type 2 diabetes mellitus. Chinese Medical Journal 2012;115:1308-11.

[R42] Santos JL, Pérez-Bravo F, Martínez JA, Montalvo D, Albala C, Carrasco E. No evidence for an association between genetic polymorphisms of beta(2)- and beta(3)-adrenergic receptor genes with body mass index in Aymara natives from Chile. Nutrition 2002;18:255-8.

[R43] Matsushita H, Kurabayashi T, Tomita M, Kato N, Tanaka K. Effects of uncoupling protein 1 and beta3-adrenergic receptor gene polymorphisms on body size and serum lipid concentrations in Japanese women. Maturitas 2003;45:39-45.

[R44] Matsushita Y, Yokoyama T, Yoshiike N, et al. The Trp(64)Arg polymorphism of the beta(3)-adrenergic receptor gene is not associated with body weight or body mass index in Japanese: a longitudinal analysis. J Clin Endocrinol Metab 2003;88:5914-20.

[R45] Zhu LY, Liu KS. The relationship between Trp64Arg mutation in the β3-adrenergic receptor gene and endothelial dysfunction in overweight and obesity subjects. Chin J Cardiol 2003; 31:118-21.

[R46] Tan N, Li L, Chen JY et al. Relationship between β3-adrenergic Trp64Arg mutation and coronary heart disease in Chinese. Chin J Cardiol. 2003;31:38-41

[R47] Shiwaku K, Nogi A, Anuurad E, et al. Difficulty in losing weight by behavioral intervention for women with Trp64Arg polymorphism of the beta3-adrenergic receptor gene. Int J Obes Relat Metab Disord 2003;27:1028-36

[R48] Okumura K, Matsui H, Ogawa Y, et al. The polymorphism of the beta3-adrenergic receptor gene is associated with reduced low-density lipoprotein particle size. Metabolism 2003;52:356-61.

[R49] Arashiro R, Katsuren K, Fukuyama S, Ohta T. Effect of Trp64Arg mutation of the beta3-adrenergic receptor gene and C161T substitution of the peroxisome proliferator activated receptor gamma gene on obesity in Japanese children. Pediatr Int 2003;45:135-41.

[R50] Kim OY, Cho EY, Park HY, Jang Y, Lee JH. Additive effect of the mutations in the beta3-adrenoceptor gene and UCP3 gene promoter on body fat distribution and glycemic control after weight reduction in overweight subjects with CAD or metabolic syndrome. Int J Obes Relat Metab Disord 2004;28:434-41.

[R51] Gao CR, Zhou DJ, Mei XB. The relationships between β3-adrenergic receptor gene polymorphism and macrovascular and microvascular complications in T2DM patients. Chin J Endocrinol Metab 2002;18:268-9.

[R52] Ramis JM, González-Sánchez JL, Proenza AM, et al. The Arg64 allele of the beta 3-adrenoceptor gene but not the -3826G allele of the uncoupling protein 1 gene is associated with increased leptin levels in the Spanish population. Metabolism 2004;53:1411-6.

[R53] Erhardt E, Czakó M, Csernus K, Molnár D, Kosztolányi G. The frequency of Trp64Arg polymorphism of the beta3-adrenergic receptor gene in healthy and obese Hungarian children and its association with cardiovascular risk factors. Eur J Clin Nutr 2005;59:955-9.

[R54] Zuo H, Zhai CK, Jiang L et al. Study on β3-adrenergic receptor gene polymorphism and effect of dietary intervention in community hyperglycemia population. Chin J Public Health 2005;21:807-9.

[R55] Tan H, Chen FR, Tang QD, Liu L. The study of correlation of β3-adrenoceptor gene polymorphism and hypertension. The Journal of Practical Medicine 2005;21:35-6.

[R56] Miyaki K, Sutani S, Kikuchi H, Takei I, Murata M, Watanabe K, Omae K. Increased risk of obesity resulting from the interaction between high energy intake and the Trp64Arg polymorphism of the beta3-adrenergic receptor gene in healthy Japanese men. J Epidemiol 2005;15:203-10.

[R57] Pérez-Bravo F, Echiburú B, Maliqueo M, Santos JL, Sir-Petermann T. Tryptophan 64 --> arginine polymorphism of beta-3-adrenergic receptor in Chilean women with polycystic ovary syndrome. Clin Endocrinol (Oxf) 2005;62:126-31.

[R58] Lee JS, Kawakubo K, Inoue S, Akabayashi A. Effect of β(3)-adrenergic receptor gene polymorphism on body weight change in middle-aged, overweight women. Environ Health Prev Med 2006;11:69-74.

[R59] Kim K, Lee S, Lee S, et al. Comparison of body fat distribution and blood lipid profiles according to Trp64Arg polymorphism for the beta 3-adrenergic receptor gene in Korean middle-aged women. J Nutr Sci Vitaminol (Tokyo) 2006;52:281-6.

[R60] Yuan M, Ohishi M, Ito N, et al. Genetic influences of beta-adrenoceptor polymorphisms on arterial functional changes and cardiac remodeling in hypertensive patients. Hypertens Res 2006;29:875-81.

[R61] Tamaki S, Nakamura Y, Tabara Y, et al. Relationship between metabolic syndrome and Trp64arg polymorphism of the beta-adrenergic receptor gene in a general sample: the Shigaraki study. Hypertens Res 2006;29:891-6.

[R62] Eshraghi P, Hedayati M, Daneshpour MS, Mirmiran P, Azizi F. Association of body mass index and Trp64Arg polymorphism of the beta3-adrenoreceptor gene and leptin level in Tehran Lipid and Glucose Study. Br J Biomed Sci 2007;64:117-20.

[R63] Klass DM, Lauer N, Hay B, Kratzer W, Fuchs M; EMIL Study Group. Arg64 variant of the beta3-adrenergic receptor is associated with gallstone formation. Am J Gastroenterol 2007;102:2482-7.

[R64] Li Q, Xu JD, Zhang Y, et al. Effects of Variation of the β3-adrenergic-receptor and Uncoupling Protein-2 Gene Polymorphism on Children Simple Obesity. Chin J Sch Health 2007;28:249-52.

[R65] Jiang XY, Hou XG, Hou WK, Ren JM, Chen L. β3-adrenoreceptor Trp64Arg polymorphism in diabetic peripheral neuropathy. Chin J Diabetes 2007;15:526-7.

[R66] Chen W, Srinivasan SR, Boerwinkle E, Berenson GS. Beta-adrenergic receptor genes are associated with arterial stiffness in black and white adults: the Bogalusa Heart Study. Am J Hypertens 2007;20:1251-7.

[R67] Piérola J, Barceló A, de la Peña M, et al. beta3-Adrenergic receptor Trp64Arg polymorphism and increased body mass index in sleep apnoea. Eur Respir J 2007;30:743-7.

[R68] Bracale R, Pasanisi F, Labruna G, et al. Metabolic syndrome and ADRB3 gene polymorphism in severely obese patients from South Italy. Eur J Clin Nutr 2007;61:1213-9.

[R69] de Luis DA, Gonzalez Sagrado M, Aller R, Izaola O, Conde R. Influence of the Trp64Arg polymorphism in the beta 3 adrenoreceptor gene on insulin resistance, adipocytokine response, and weight loss secondary to lifestyle modification in obese patients. Eur J Intern Med 2007;18:587-92.

[R70] Ueno T, Takahashi Y, Matsumoto T, et al. Postprandial plasma lipid levels are influenced by the interaction of functional polymorphisms in the microsome triglyceride transfer protein and beta3 adrenergic receptor genes. Med Sci Monit 2007;13:BR112-8.

[R71] Dunajska K, Lwow F, Tworowska U, Jedrzejuk D, Milewicz A. Relationship of beta(3)-adrenergic receptor polymorphism with metabolic syndrome and oxidative stress parameters in postmenopausal women. Endokrynol Pol. 2007;58:201-6.

[R72] Yamada K, Takezawa J, Morita A, Matsumura, Watanabe S. DNA polymorphism of obese people in Saku Control Obesity Program (SCOP). Anti-Aging Medicine 2007;4:63-9.

[R73] Tsuzaki K, Kotani K, Fujiwara S, et al. The Trp64Arg polymorphism of the beta3-adrenergic receptor gene is associated with increased small dense low-density lipoprotein in a rural Japanese population: the Mima study. Metabolism 2007;56:1689-93.

[R74] de Luis DA, Aller R, Izaola O, Gonzalez-Sagrado M, Conde R. Relation of Trp64Arg polymorphism of beta3-adrenoreceptor gene with cardiovascular risk factors in presurgical morbidly obese patients. Arch Med Res 2008;39:791-5.

[R75] Kotani K, Sakane N, Kurozawa Y, et al. Polymorphism of Trp64Arg in beta3-adrenergic receptor gene and serum LDL-cholesterol concentrations in healthy Japanese. Ann Clin Biochem 2008;45:313-5.

[R76] Chen H, Tang Y, Luo JW, et al. Relationship between β3-AR gene T190C polymorphism and the first cardiovascular and cerebrovascular events in hypertensive patient. Chin J Clinicians 2008;2:1378-85.

[R77] Zhu LY, Liu YH, Wang XH. Relationship Between Trp64Arg Mutation in β3-adrenergic Receptor Gene and Weight Reduction in Simple Obesity. Practical Preventive Medicine 2008;15:660-2.

[R78] Gjesing AP, Andersen G, Borch-Johnsen K, Jørgensen T, Hansen T, Pedersen O. Association of the beta3-adrenergic receptor Trp64Arg polymorphism with common metabolic traits: studies of 7605 middle-aged white people. Mol Genet Metab 2008;94:90-7.

[R79] Nonen S, Yamamoto I, Liu J, et al. Adrenergic beta1 receptor polymorphism (Ser49Gly) is associated with obesity in type II diabetic patients. Biol Pharm Bull 2008;31:295-8.

[R80] Hamada T, Kotani K, Higashi A, et al. Lack of association of the Trp64Arg polymorphism of beta3-adrenergic receptor gene with energy expenditure in response to caffeine among young healthy women. Tohoku J Exp Med 2008;214:365-70.

[R81] Dunajska K, Lwow F, Milewicz A, et al. beta(3)-adrenergic receptor polymorphism and metabolic syndrome in postmenopausal women. Gynecol Endocrinol 2008;24:133-8.

[R82] Zafarmand MH, van der Schouw YT, Grobbee DE, de Leeuw PW, Bots ML. T64A polymorphism in beta3-adrenergic receptor gene (ADRB3) and coronary heart disease: a case-cohort study and meta-analysis. J Intern Med 2008;263:79-89.

[R83] de Luis DA, González Sagrado M, Aller R, Izaola O, Conde R. Influence of Trp64Arg polymorphism of beta 3-adrenoreceptor gene on insulin resistance, adipocytokines and weight loss secondary to two hypocaloric diets. Ann Nutr Metab 2009;54:104-10.

[R84] Zhu LY, Hu LY, Li XL, et al. A Seven-year Follow-up Study of the Relationship Between Trp64Arg Mutation in the β3 adrenergic Receptor Gene and Cardiovascular Disease Risk Factors in Obese Subjects. Clinical Misdiagnosis & Mistherapy 2009;22:22-4.

[R85] Yu B, Qi Y, Dou GW, Wu ZH. No aasociation between ADRB3 gene Trp64Arg polymorphism and Japanese national essential hypertensive patients. Journal of Southw est Unive rsity 2009;31:64-8.

[R86] Zhang JH, Xu PR, Li L, Chang ZS. Study on Beta3-adrenergic receptor gene polymorphism in Karzak obese children in xin jiang. Chinese Journal of Practical Pediatrics 2009;24:34-40.

[R87] Yang M, Huang Q, Wu J, et al. Effects of UCP2 -866 G/A and ADRB3 Trp64Arg on rosiglitazone response in Chinese patients with Type 2 diabetes. Br J Clin Pharmacol 2009;68:14-22.

[R88] Li HH, Yang M, Yu M, Zhou HH, Liu ZQ. Effects of β3-AR Trp64Arg gene polymorphism on the efficacy of rosiglitazone in patients with T2DM. Chinese Pharmacological Bulletin 2009;25:317-21.

[R89] Chen YZ, Li L, Qu N, Li DH. Relationship between β3-adrenergic Trp64Arg Mutation and Instent Restenosis after CHD Stenting. Chinese General Practice. 2010;13:3984-6.

[R90] Zhu LY, Hu LY, Li XL, et al. Relationship between Trp64Arg mutation in the β3-adrenergic receptor gene and metabolic syndrome: a seven-year follow-up study. Chin Med J (Engl) 2010;123:2375-8.

[R91] Kim SM, Oh SD, Jung IG, et al. Distribution of the Trp64Arg polymorphism in the ß3-adrenergic receptor gene in athletes and its influence on cardiovascular function. Kardiol Pol 2010;68:920-6.

[R92] de Luis DA, Ballesteros M, Ruiz E, et al. Polymorphism Trp64Arg of beta 3 adrenoreceptor gene: allelic frequencies and influence on insulin resistance in a multicenter study of Castilla-León. Nutr Hosp 2010;25:299-303.

[R93] Morcillo S, Rojo-Martínez G, Martín-Núñez GM, et al. Trp64Arg polymorphism of the ADRB3 gene predicts hyperuricemia risk in a population from southern Spain. J Rheumatol 2010;37:417-21.

[R94] Peng AN, Yang SP, Zhang B. Detection and Significance of Trp64Arg Mutation of β3-AR in Preschool Children with Simple Obesity. Acta Med Univ Sci Technol Huazhong 2010;39:868-71.

[R95] Genelhu VA, Francischetti EA, Duarte SF, et al. Beta3-adrenergic receptor polymorphism is related to cardiometabolic risk factors in obese Brazilian subjects. Genet Mol Res 2010;9:1392-7.

[R96] Tsunekawa K, Yanagawa Y, Aoki T, et al. Association between accumulation of visceral fat and the combination of β3 adrenergic receptor Trp64Arg, β2 adrenergic receptor Arg16Gly and uncoupling protein 1 -3826A>G polymorphisms detected by Smart Amplification Process 2. Endocr J 2011;58:1079-86.

[R97] Mirrakhimov AE, Kerimkulova AS, Lunegova OS, et al. An association between TRP64ARG polymorphism of the B3 adrenoreceptor gene and some metabolic disturbances. Cardiovasc Diabetol 2011;10:89.

[R98] Malik SG, Saraswati MR, Suastika K, Trimarsanto H, Oktavianthi S, Sudoyo H. Association of beta3-adrenergic receptor (ADRB3) Trp64Arg gene polymorphism with obesity and metabolic syndrome in the Balinese: a pilot study. BMC Res Notes 2011;4:167.

[R99] Iwamoto Y, Ohishi M, Yuan M, et al. β-Adrenergic receptor gene polymorphism is a genetic risk factor for cardiovascular disease: a cohort study with hypertensive patients. Hypertens Res 2011;34:573-7.

[R100] Kwon DH, Bose S, Song MY, et al. Efficacy of Korean Red Ginseng by Single Nucleotide Polymorphism in Obese Women: Randomized, Double-blind, Placebo-controlled Trial. J Ginseng Res 2012;36:176-89.

[R101] Zhang XY, Wei FJ, Xu L, Yin SY. Association of Beta 3 Adrenergic Receptor Gene Polymorphism and Essential Hypertension in Mogolian Population. Chinese Journal Of Integrative Medicine On Cardio-/Cerebrovascular Disease 2012;10:658-60.

[R102] de Luis DA, Aller R, Izaola O, González Sagrado M, Conde R. Association of -55CT polymorphism of UCP3 gene with fat distribution, cardiovascular risk factors and adipocytokines in patients with Type 2 diabetes mellitus. J Endocrinol Invest 2012;35:625-8.

[R103] de Luis DA, Aller R, Izaola O, Conde R, Eiros Bouza JM. Genetic variation in the beta 3-adrenoreceptor gene (Trp64Arg polymorphism) and its influence on anthropometric parameters and insulin resistance under a high monounsaturated versus a high polyunsaturated fat hypocaloric diet. Ann Nutr Metab 2013;62:303-9.

[R104] Hameed I, Masoodi SR, Afroze D, Naykoo NA, Bhat RA, Ganai BA. Trp homozygotes at codon 64 of ADRB3 gene are protected against the risk of type 2 diabetes in the Kashmiri population. Genet Test Mol Biomarkers 2013;17:775-9.

[R105] Oguri K, Tachi T, Matsuoka T. Visceral fat accumulation and metabolic syndrome in children: the impact of Trp64Arg polymorphism of the beta3-adrenergic receptor gene. Acta Paediatr 2013;102:613-9.

[R106] Zhang XL. Association of β3AR Gene Trp64Arg Locus Polymorphism and Effectiveness of Exercise Rehabilitation on Male Adolescents of Metabolic Syndrome. Journal of Shenyang Sport University 2014;33:84-8.

[R107] Kumar S, Mishra A, Srivastava A, Mittal T, Garg N, Mittal B. Significant role of ADRB3 rs4994 towards the development of coronary artery disease. Coron Artery Dis 2014;25:29-34.

[R108] Brondani LA, Duarte GC, Canani LH, Crispim D. The presence of at least three alleles of the ADRB3 Trp64Arg (C/T) and UCP1-3826A/G polymorphisms is associated with protection to overweight/obesity and with higher high-density lipoprotein cholesterol levels in Caucasian-Brazilian patients with type 2 diabetes. Metab Syndr Relat Disord 2014;12:16-24.

[R109] Iemitsu M, Fujie S, Murakami H, et al. Higher cardiorespiratory fitness attenuates the risk of atherosclerosis associated with ADRB3 Trp64Arg polymorphism. Eur J Appl Physiol 2014;114:1421-8.

[R110] Wang T, Zhang Y, Ma J, Feng Z, Niu K, Liu B. Additive effect of polymorphisms in the β2-adrenoceptor and NADPH oxidase p22 phox genes contributes to the loss of estimated glomerular filtration rate in Chinese. Clin Exp Pharmacol Physiol 2014;41:657-62.

[R111] Yoshihara A, Sugita N, Iwasaki M, Miyazaki H, Nakamura K. The interaction between beta-3 adrenergic receptor polymorphism and obesity to periodontal disease in community-dwelling elderly Japanese. J Clin Periodontol 2014;41:460-6.

[R112] Hui ZG, Zhou XW, Li WT, Sun JH, Hou NN, Sun HX. Relationship between gene polymorphism of beta 3-adrenergic receptor and diabetic foot. Int J Endocrinol Metab 2014;34:138-40.

[R113] de Luis DA, Aller R, Izaola O, de la Fuente B, Romero E. GENETIC VARIATION IN THE BETA-3-ADRENORECEPTOR GENE (TRP64ARG POLYMORPHISM) AND THEIR INFLUENCE ON ANTHROPOMETRIC PARAMETERS AND INSULIN RESISTANCE AFTER A HIGH PROTEIN/LOW CARBOHYDRATE VERSUS A STANDARD HYPOCALORIC DIET. Nutr Hosp 2015;32:487-93.

[R114] Chen Y, Wang X, Shen Z, et al. Effect of the beta-3 adrenergic receptor Trp64Arg and uncoupling protein 1-3826 A>G genotypes on lipid and apolipoprotein levels in overweight/obese and non-obese Chinese subjects. Lipids Health Dis 2015;20;14:34.

[R115] Verdi H, Tulgar Kınık S, Yılmaz Yalçın Y, Muratoğlu Şahin N, Yazıcı AC, Ataç FB. β-3AR W64R Polymorphism and 30-Minute Post-Challenge Plasma Glucose Levels in Obese Children. J Clin Res Pediatr Endocrinol 2015;7:7-12.

[R116] Liu JL, Zhang B, Li M, et al. Study on relationship between Trp64Arg polymorphism of B3-adrenergic receptor gene and obesity and blood lipids. Natl Med J China 2015;95:1558-62.

[R117] Dong CM. Correlation of β3-adrenergic receptor gene polymorphism and aerobic exercise intervention in patients with metabolic syndrome. Chinese Journal of Gerontology 2016;36:4451-4.

[R118] De Luis Román DA, Primo D, Izaola O, Aller R. Relation of Trp64Arg polymorphism of beta 3 adrenoreceptor gene with metabolic syndrome and insulin resistance in obese women. Nutr Hosp. 2017;34:383-388.

[R119] Milano-Gai GE, Furtado-Alle L, Mota J, et al. 12-Week aerobic exercise and nutritional program minimized the presence of the 64Arg allele on insulin resistance. J Pediatr Endocrinol Metab 2018;31:1033-42.

[R120] Jesus ÍC, Alle LF, Munhoz EC, et al. Trp64Arg polymorphism of the ADRB3 gene associated with maximal fat oxidation and LDL-C levels in non-obese adolescents. J Pediatr (Rio J) 2018;94:425-31.

[R121] Daghestani M, Daghestani M, Daghistani M, et al. ADRB3 polymorphism rs4994 (Trp64Arg) associates significantly with bodyweight elevation and dyslipidaemias in Saudis but not rs1801253 (Arg389Gly) polymorphism in ARDB1. Lipids Health Dis 2018;17:58.

[R122] Grygiel-Górniak B, Kaczmarek E, Mosor M, Przysławski J, Nowak J. Gene-diet-related factors of hyperglycaemia in postmenopausal women. J Appl Genet 2018;59:169-77.
